# Integrated genomic and clinical modeling for prognostic assessment of radiotherapy response in rectal neoplasms

**DOI:** 10.1515/biol-2025-1199

**Published:** 2025-12-30

**Authors:** Chen Li, Jie Zhang, Shuangxiang Lin, Xinhong Wang, Zhongjian Ju

**Affiliations:** China CDC Key Laboratory of Radiological Protection and Nuclear Emergency, Chinese Center for Disease Control and Prevention, National Institute for Radiological Protection, Beijing, 100088, P.R. China; University of Science and Technology of China, Hefei, 230026, Anhui, P.R. China; High Magnetic Field Laboratory, Key Laboratory of High Magnetic Field and Ion Beam Physical Biology, Chinese Academy of Sciences, Hefei, P.R. China; Anhui Province Key Laboratory of Environmental Toxicology and Pollution Control Technology, Hefei Institutes of Physical Science, Chinese Academy of Sciences, Hefei, Anhui, 230031, P.R. China; Department of Radiology, The Second Affiliated Hospital, Zhejiang University, School of Medicine, Hangzhou, 310009, P.R. China; Department of Radiation Oncology, The First Medical Center, People’s Liberation Army General Hospital, Beijing, 100853, P.R. China; LIST, Key Laboratory of New Generation Artificial Intelligence Technology and Its Interdisciplinary Applications (Southeast University), Ministry of Education, Nanjing 210096, China

**Keywords:** bioinformatic analysis, drug resistance and mitophagy-related genes, immune infiltration, prognostic risk model, rectal cancer radiotherapy

## Abstract

The aim of this study is to develop a prognostic model for evaluating radiotherapy response in patients diagnosed with rectal neoplasms by integrating genomic and clinical data. Publicly accessible datasets from The Cancer Genome Atlas and Gene Expression Omnibus were analyzed to identify differentially expressed genes associated with drug resistance and mitophagy. Functional enrichment analyses were conducted to investigate relevant biological pathways. A prognostic risk model was constructed using least absolute shrinkage and selection operator regression and validated via receiver operating characteristic (ROC) curve analysis and Kaplan–Meier survival analysis. The final model incorporated 15 genes selected from an initial set of 121 differentially expressed genes and demonstrated moderate to high predictive accuracy for 1-, 2-, and 3-year overall survival (area under the ROC curve: 0.70–0.90). Kaplan–Meier analysis revealed statistically significant differences in survival outcomes between high-risk and low-risk patient groups. Pathway enrichment analysis indicated that the selected genes were involved in actin cytoskeleton reorganization and antiviral immune responses. Differential expression of key genes within the model was confirmed through quantitative polymerase chain reaction assays. The resulting prognostic model enhances understanding of the molecular mechanisms underlying radiotherapy response in rectal neoplasms and may support individualized therapeutic decision-making in clinical oncology.

## 1 Introduction

Rectal cancer remains one of the leading causes of cancer-related mortality globally and presents significant clinical challenges due to the heterogeneity in patient responses to radiotherapy [1]. Although radiotherapy is a cornerstone in the management of locally advanced rectal cancer, variability in therapeutic outcomes underscores the need for reliable biomarkers to support individualized treatment strategies. Despite recent advances in genomic profiling, the molecular mechanisms underlying radiotherapy response in rectal cancer are not yet fully understood [2].

A critical gap in the current research landscape is the absence of robust and clinically applicable biomarkers capable of accurately predicting radiotherapy outcomes, in rectal malignancies. In this context, increasing attention has been directed toward two phenotypic features – drug resistance and mitophagy – that significantly influence tumor progression and treatment responsiveness [3], 4].

Drug resistance refers to the ability of malignant cells to evade the cytotoxic effects of anticancer agents, often resulting in treatment failure and disease recurrence. This resistance is multifactorial, involving genetic mutations, epigenetic modifications, and dysregulation of signaling pathways that collectively contribute to therapeutic insensitivity [3]. Conversely, mitophagy, the selective autophagic degradation of mitochondria, serves as a key regulator of cellular homeostasis by eliminating damaged mitochondria and preserving metabolic equilibrium [4]. The role of mitophagy in oncogenesis is context-dependent, with evidence supporting both tumor-suppressive and tumor-promoting functions depending on disease stage and microenvironmental conditions.

The interaction between drug resistance and mitophagy in rectal cancer remains insufficiently elucidated. However, emerging data suggest that both processes may serve as important biomarkers and therapeutic targets. Altered expression of mitophagy-related genes has been associated with chemoresistance in multiple malignancies, while modulation of drug resistance pathways has shown potential to enhance radiotherapy efficacy [5], [6], [7], [8].

Given the clinical significance of these phenotypes, further investigation is warranted to define their functional relevance in rectal cancer. Such studies may inform the development of individualized treatment strategies. Drug resistance and mitophagy, in particular, hold promise as biomarkers for predicting radiotherapy response, contributing to the advancement of precision medicine in oncology.

Although substantial efforts have been made to characterize the genomic landscape of rectal cancer, particularly regarding gene expression profiles linked to treatment response, the specific contributions of drug resistance- and mitophagy-related genes (DRMRGs) in modulating radiotherapy outcomes remain poorly characterized. Leveraging advanced bioinformatics and large-scale genomic datasets, the present study aimed to identify DRMRGs with potential prognostic value in the context of radiotherapy.

This study employed an integrative approach, informed by a comprehensive review of observational studies, experimental models, and clinical trial data. A key limitation in the existing literature is the lack of predictive models that incorporate both genomic and clinical variables to predict individual radiotherapy responses in rectal cancer. This shortcoming is particularly relevant given the biological heterogeneity of rectal cancer and the variability in therapeutic outcomes across patient populations.

To address this gap, a prognostic risk model was developed by integrating DRMRG expression data with clinically relevant variables. This model aims to improve the understanding of molecular and clinical factors influencing radiotherapy efficacy and to support refined patient stratification based on predicted treatment response. The integration of genomic features with clinical parameters is intended to guide the development of individualized therapeutic strategies and optimize treatment planning.

This work contributes to the field of precision oncology by facilitating the identification of molecular biomarkers capable of informing personalized treatment decisions. The proposed model represents a step forward in delivering targeted, effective, and patient-centered care for individuals with rectal cancer.

## 2 Materials and methods

### 2.1 Technology roadmap

Gene expression datasets GSE150082 and GSE35452, comprising 16 radiotherapy-responsive and 24 non-responsive samples, were integrated and normalized prior to analysis. Immune cell infiltration was evaluated using the CIBERSORT algorithm, with a focus on DRMRGs. Pathway-level functional enrichment was assessed through gene set variation analysis (GSVA) and gene set enrichment analysis (GSEA).

Functional annotation of differentially expressed genes was conducted using enrichment analyses based on the Kyoto Encyclopedia of Genes and Genomes (KEGG) and Gene Ontology (GO) databases. A prognostic risk model was subsequently developed by incorporating somatic mutation (SM) data and copy number variation (CNV) profiles, enabling stratification of patients into distinct risk categories.

**Figure 1:**
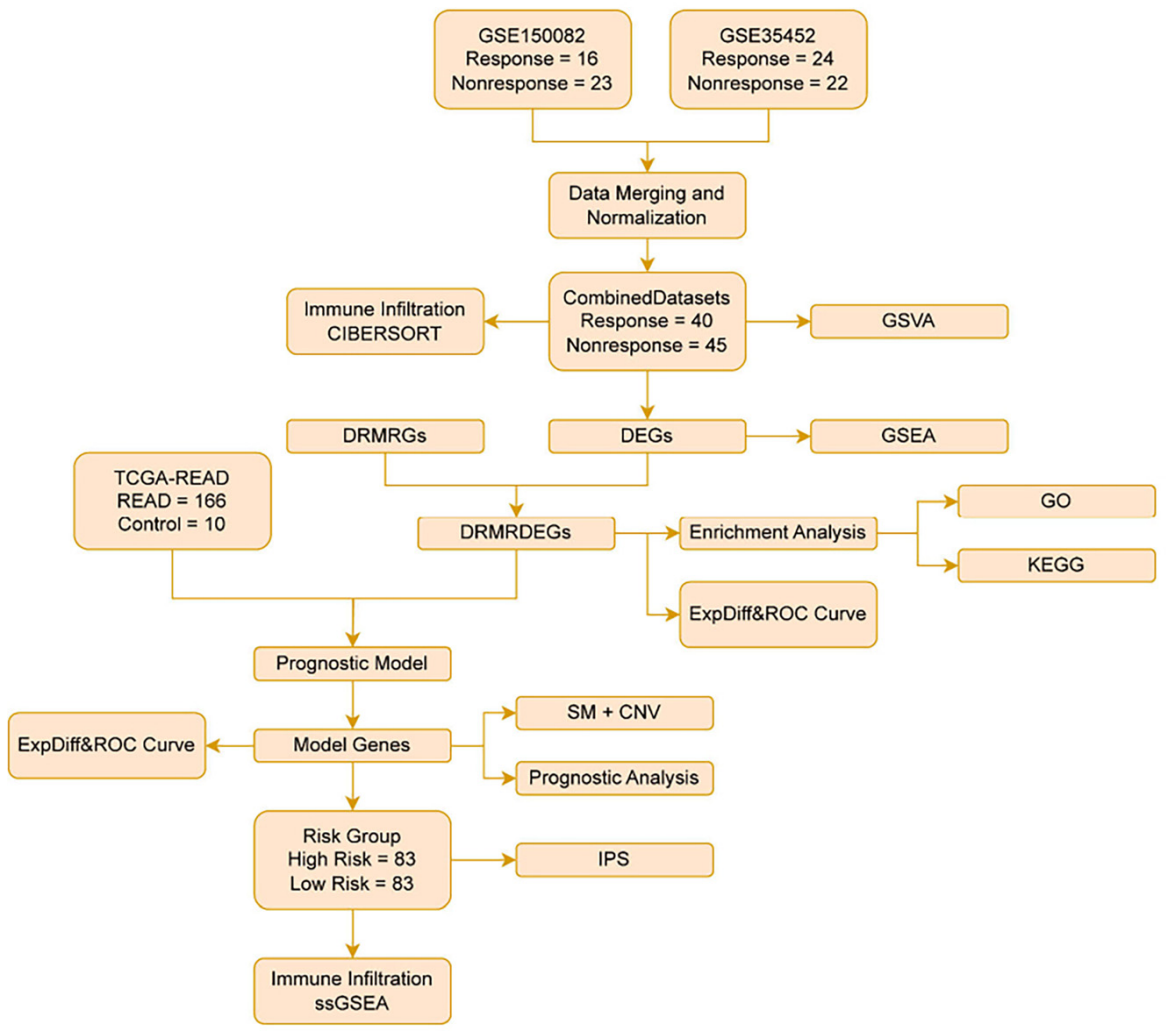
Flow chart for the comprehensive analysis of DRMRDEGs. TCGA, the cancer genome atlas; READ, rectal adenocarcinoma; GSVA, gene set variation analysis; DEGs, differentially expressed genes; DRMRGs, drug resistance & mitophagy-related genes; GSEA, gene set enrichment analysis; DRMRDEGs, drug resistance- and mitophagy-related differentially expressed genes; GO, gene ontology; KEGG, kyoto encyclopedia of genes and genomes; SM, somatic mutation; CNV, copy number variations; IPS, immunophenoscore; ssGSEA, single-sample gene-set enrichment analysis; ROC, receiver operating characteristic.

In addition, single-sample gene-set enrichment analysis (ssGSEA) was applied to characterize immune cell infiltration patterns, providing a detailed assessment of the tumor immune microenvironment and its relationship with patient prognosis (Figure 1). All datasets utilized in this study are publicly available, de-identified, and were previously reviewed and approved by the respective institutional ethics committees responsible for data generation, in accordance with the Declaration of Helsinki.

### 2.2 Data download

The rectal adenocarcinoma (READ) dataset was obtained from The Cancer Genome Atlas (TCGA) and processed using the R package TCGAbiolinks [9]. Following the exclusion of samples lacking prognostic information, a total of 166 READ samples with prognostic data and 10 control samples were included. Raw sequencing data in counts format were normalized by conversion to fragments per kilobase of transcript per million mapped reads (FPKM). Associated clinical data were retrieved from the UCSC Xena platform and are summarized in Table S1 [10].

Datasets GSE150082 and GSE35452 were downloaded from the Gene Expression Omnibus (GEO) database (https://www.ncbi.nlm.nih.gov/geo/, with a focus on rectal cancer samples subjected to radiotherapy [11], [12], [13]. Both datasets consist of *Homo sapiens*-derived rectal tissue samples from patients who underwent radiotherapy. GSE150082 (platform: GPL13497) included 16 radiotherapy-responsive and 23 non-responsive samples. GSE35452 (platform: GPL570) contained 24 responsive and 22 non-responsive samples. All available samples from both datasets were included in downstream analysis, as detailed in Table S2.

Drug resistance-related genes (DRRGs) were identified using the GeneCards database (https://www.genecards.org/) with the search term “Drug Resistance,” yielding 7,774 protein-coding genes. An additional 103 DRRGs were obtained from the MSigDB database (https://www.gsea-msigdb.org/gsea/msigdb) [14], 15], specifically, from the gene sets: KESHELAVA_MULTIPLE_DRUG_RESISTANCE, YAGUE_PRETUMOR_DRUG_RESISTANCE_DN, and YAGUE_PRETUMOR_DRUG_RESISTANCE_UP. After merging and removing duplicate entries, a total of 7,826 unique DRRGs were compiled.

Mitophagy-related genes (MRGs) were collected using a similar approach. The GeneCards database was queried with the term “Mitophagy,” resulting in 4,878 protein-coding genes. An additional 29 genes were curated from a PubMed-based literature search (https://pubmed.ncbi.nlm.nih.gov/) [16]. Following deduplication, 4,878 unique MRGs were retained. The intersection of the DRRG and MRG lists yielded 3,143 DRMRGs.

To correct for batch effects between GSE150082 and GSE35452, the *sva* R package was employed, generating a harmonized dataset referred to as the combined dataset. [17]. This integrated dataset included 40 radiotherapy-responsive and 45 non-responsive samples. The resulting gene expression matrix was then intersected with the TCGA-READ gene expression data to retain only overlapping genes for integrative analysis.

Standardization, probe annotation, and normalization of gene expression data were conducted using the limma R package [18]. Principal component analysis (PCA) was performed both before and after batch effect correction to evaluate the effectiveness of the harmonization process [18], 19]. PCA was also used to reduce the dimensionality of the high-throughput data, allowing extraction of principal components for visualization in two- or three-dimensional space.

### 2.3 DRMRDEGs associated with colorectal cancer (CRC)

Within the combined dataset, samples were classified into radiotherapy-responsive and non-responsive groups. Differential gene expression analysis between the two groups was performed using the R package limma. Differentially expressed genes (DEGs) were identified based on the criteria of absolute log_2_ fold change (|logFC|) > 0 and a *p*-value < 0.05. Genes with logFC > 0 and *p*-value < 0.05 were classified as upregulated, whereas those with logFC < 0 and *p*-value < 0.05 were classified as downregulated. The results of the differential expression analysis were visualized using a volcano plot generated with the R package ggplot2.

To identify drug resistance- and mitophagy-related differentially expressed genes (DRMRDEGs) associated with READ, all DEGs meeting the specified thresholds were intersected with the previously identified DRMRGs. The overlapping genes were visualized using a Venn diagram to highlight the DRMRDEGs. A heatmap was subsequently generated using the R package pheatmap to depict the expression profiles of these intersected genes across the sample groups.

### 2.4 Construction of prognostic risk model for rectal cancer

To construct the prognostic risk model based on the TCGA-READ dataset, the R package glmnet was used with the parameter setting family = “cox” [20]. DRMRDEGs were analyzed using least absolute shrinkage and selection operator (LASSO) regression, with the random seed set to 500 for reproducibility in model gene selection.

LASSO regression, a penalized linear regression method, incorporates a penalty term (lambda × the absolute value of the slope) to reduce overfitting and improve the model’s generalizability. The output of the LASSO regression analysis was visualized through a prognostic risk model diagram and a coefficient trajectory plot.

Subsequently, a LASSO-derived RiskScore (hereafter referred to as RiskScore) was calculated using the regression coefficients associated with the selected genes, based on the following formula:

$$\text{riskScore}=\underset{i}{\sum }\text{Coefficient }\left({\text{gene}}_{i}\right)*\text{mRNA Expression }\left({\text{gene}}_{i}\right)$$

### 2.5 Prognostic analysis of prognostic risk model for rectal cancer

Time-dependent receiver operating characteristic (ROC) curve analysis is commonly employed to evaluate the predictive performance of prognostic models, including the determination of optimal thresholds and the exclusion of suboptimal alternatives [21]. In this study, time-dependent ROC curves were generated using the R package survivalROC. The area under the curve (AUC) was calculated based on the RiskScore and overall survival (OS) data to predict 1-, 2-, and 3-year survival probabilities for patients with READ in the TCGA-READ dataset.

The AUC value of a ROC curve ranges from 0.5 to 1.0, with values closer to 1.0 indicating superior discriminatory performance. An AUC greater than 0.5 indicates a positive association between gene expression and event occurrence. AUC values between 0.5 and 0.7 indicate low accuracy, values between 0.7 and 0.9 indicate moderate accuracy, and values above 0.9 are considered highly accurate.

To assess differences in OS between high- and low-risk groups among READ samples from the TCGA-READ dataset, Kaplan–Meier (KM) survival curves were generated using the R package survival, with group classification determined based on the RiskScore [22].

Univariate and multivariate Cox regression analyses were conducted to examine the prognostic relevance of the RiskScore along with clinical variables. The results were visualized using forest plots to display hazard ratios and confidence intervals for each variable included in the analysis.

A nomogram was constructed using the R package rms based on the results of the multivariate Cox regression analysis [23]. This graphical tool aligns independent prognostic factors along separate axes to estimate survival probabilities at specified time points, thereby facilitating individualized survival prediction.

To assess the predictive performance of the model, calibration curves were plotted to compare predicted survival probabilities with actual outcomes. These curves were used to assess the agreement between model predictions and observed survival data, thereby assessing the calibration and discrimination capabilities of the prognostic risk model based on the RiskScore.

### 2.6 Differential expression verification and ROC curve analysis

To further assess the expression differences of DRMRDEGs between radiotherapy-responsive and non-responsive groups in the combined datasets, comparative expression plots were generated using the corresponding gene expression profiles. The R package pROC was employed to construct ROC curves and calculate the AUC values, thereby assessing the diagnostic potential of DRMRDEG expression levels in predicting radiotherapy response in READ.

Subsequently, samples from the TCGA-READ dataset were stratified into high- and low-risk groups based on the median value of the RiskScore derived from the prognostic risk model. Expression differences of the model-selected genes between these two risk groups were then analyzed, and comparative group plots were created to visualize the results. ROC curve analysis for the model genes was again conducted using the R package pROC, and the corresponding AUC values were calculated to evaluate the diagnostic efficacy of gene expression levels in relation to READ occurrence.

### 2.7 Analyses of SMs and CNVs

To analyze somatic mutations (SMs) in READ samples from the TCGA-READ dataset, the “Masked Somatic Mutation” dataset was selected as the source of SM data. Preprocessing of these data was conducted using VarScan software. The somatic mutation landscape was then visualized using the R package maftools [24].

For the analysis of copy number variations (CNVs) in the same cohort, the “Masked Copy Number Segment” data from TCGA were used. The downloaded and preprocessed CNV segments were analyzed using GISTIC2.0, applying default parameters to identify genomic regions with statistically significant alterations associated with cancer [25].

### 2.8 GO and KEGG pathway enrichment analyses

GO analysis is a widely adopted approach for functional enrichment studies involving large gene sets, encompassing three primary domains: biological processes (BP), cellular components (CC), and molecular functions (MF) [26]. The is an integrated database resource that links genomic information with biological pathways, diseases, and therapeutic agents [27].

To explore the functional characteristics of DRMRDEGs, GO and KEGG pathway enrichment analyses were performed using the R package clusterProfiler [28]. Statistical significance was defined by an adjusted *p*-value (adj. *p*) < 0.05 and a false discovery rate (FDR, or *q*-value) < 0.25. The Benjamini–Hochberg (BH) method was used for multiple testing correction to control the FDR.

### 2.9 GSEA

GSEA is a computational method used to evaluate whether members of a predefined gene set are statistically significantly enriched at the top or bottom of a ranked gene list, thereby indicating their potential contribution to a specific phenotype [29]. In this study, genes from the combined datasets were ranked according to their logFC values. GSEA was then conducted on the entire gene list using the R package clusterProfiler.

The analysis was conducted using the following parameters: the random seed was set to 2020, the number of permutations was fixed at 1,000, and the minimum and maximum number of genes per gene set were set at 10 and 500, respectively. The c2 gene set (cp.all.v2022.1. hs.symbols.gmt), representing a collection of 3,050 canonical pathways, was obtained from the Molecular Signatures Database (MSigDB) and used for the enrichment analysis [15].

Statistical significance for enrichment was defined as an adjusted *p*-value (adj. *p*) < 0.05 and an FDR or *q*-value < 0.25. Multiple testing correction was performed using the BH method to control the FDR.

### 2.10 GSVA

GSVA is a non-parametric, unsupervised analytical method used to assess the relative enrichment of predefined gene sets across different samples [30]. This approach transforms a gene expression matrix from a gene-centric format into a sample-centric enrichment score matrix, enabling the evaluation of pathway-level differences among samples. The objective is to identify pathways that are differentially enriched between distinct biological conditions.

In this study, the gene set file c2. all.v2022.2. hs.symbols.gmt was obtained from the Molecular Signatures Database (MSigDB). All gene sets within this file were included in the GSVA, which was conducted using the combined dataset to assess functional enrichment differences between the radiotherapy-responsive and non-responsive groups. Statistical significance for pathway enrichment was defined as *p*-value < 0.05.

### 2.11 Immune infiltration analysis

CIBERSORT is an analytical algorithm that uses linear support vector regression to deconvolute transcriptomic data, thereby estimating the composition and relative abundance of immune cell types within heterogeneous cell populations [31]. In this study, the CIBERSORT algorithm was applied to the combined datasets in conjunction with the LM22 signature matrix. Samples exhibiting an immune cell enrichment score greater than zero were retained, resulting in a refined immune cell infiltration matrix. The proportional distribution of immune cell types was visualized using a bar chart.

Spearman’s rank correlation coefficient was used to assess pairwise correlations among immune cell types, and the results were visualized as a correlation heatmap generated using the R package pheatmap. Additionally, correlations between model gene expression levels and immune cell proportions were evaluated using Spearman correlation. Statistically significant associations (*p*-value < 0.05) were retained and visualized using bubble plots created with the R package ggplot2.

ssGSEA was performed to quantify the relative abundance of immune cell infiltrates at the individual sample level [32]. A curated list of human immune cell types, including activated CD8^+^ T cells, activated dendritic cells, γδ T cells, natural killer cells, and regulatory T cells, was used for annotation. The resulting enrichment scores were used to generate an immune cell infiltration matrix for the TCGA-READ samples.

Differences in immune cell infiltration between high- and low-risk groups, as defined by the RiskScore, were statistically assessed, and results were visualized using group comparison plots generated with ggplot2. Spearman’s correlation analysis was also used to explore associations among immune cell subsets and between model genes and immune cell types, with statistically significant findings (*p* < 0.05) displayed using correlation heatmaps and bubble plots, respectively.

### 2.12 Immunogenicity score analysis

Immunogenicity refers to the capacity of an antigen or its epitopes to interact with antigen recognition receptors on T and B cells, thereby eliciting humoral and/or cell-mediated immune responses. Agents capable of initiating such responses are termed immunogens. The degree of immunogenicity is influenced by multiple genetic factors, including those associated with effector cells, major histocompatibility complex molecules, immunomodulatory pathways, and immunosuppressive cellular components. Recent advances in computational immunology have enabled the quantification of immunogenicity using machine learning techniques [33].

The Cancer Immunome Atlas (TCIA) database (https://tcia.at/home) provides immunophenoscores (IPS) for 20 cancer types, serving as predictive indicators of immune responsiveness to immune checkpoint inhibitors such as CTLA-4 and PD-1 [25]. In this study, IPS data corresponding to TCGA-READ samples were obtained from the TCIA database. Samples were stratified into high- and low-risk groups according to the RiskScore derived from the prognostic model. Comparative analysis of IPS values between these groups was conducted, and the distribution of IPS scores was visualized using the R package ggplot2. Statistical tests were performed to determine the significance of differences in immunogenicity between risk groups.

### 2.13 Reverse Transcription Quantitative PCR (RT–qPCR)

Total RNA was extracted using TRIzol reagent (Invitrogen), and complementary DNA (cDNA) was synthesized using the HiScript III RT SuperMix kit (Vazyme, R323-01). Quantitative real-time PCR (qPCR, with genomic DNA wiper) was performed in technical triplicates using ChamQ SYBR qPCR Master Mix (Vazyme, Q311-03) on a LightCycler 96 System (Roche). Melt curve analysis was conducted to verify amplification specificity.

The experiment included three independent biological replicates (*n* = 3). Gene expression levels were normalized to the geometric mean of two validated reference genes, *β-actin* and *GAPDH*, which were confirmed to be stably expressed under the experimental conditions. Relative expression was calculated using the 2^(−ΔΔCT)^ method.

Statistical significance for comparisons between two groups was determined using Student’s t-test. For comparisons among multiple groups, one-way ANOVA followed by Tukey’s post hoc test was applied. Data are reported as mean ± standard deviation (SD), and a *p*-value < 0.05 was considered statistically significant.

### 2.14 Statistical analysis

All data processing and statistical analyses were conducted using R software (Version 4.3.0). For comparisons involving continuous variables between two groups, independent Student’s t-tests were used to assess statistical significance for normally distributed data, unless otherwise specified. For non-normally distributed variables, the Mann–Whitney *U* test (also referred to as the Wilcoxon rank-sum test) was applied. Comparisons across three or more groups were conducted using the Kruskal–Wallis test.

Spearman’s rank correlation analysis was used to compute correlation coefficients between molecular variables. All *p*-values were two-sided unless otherwise noted, and a *p*-value < 0.05 was considered indicative of statistical significance.

## 3 Results

### 3.1 Merging rectal cancer datasets

Batch effects present in the rectal adenocarcinoma READ radiotherapy datasets GSE150082 and GSE35452 were corrected using the R package sva, resulting in the generation of the combined datasets. To evaluate the effectiveness of batch effect removal, expression value distributions before and after correction were visualized using boxplots (Figure S1A–B). Additionally, PCA plots were used to compare the distributions of low-dimensional features before and after batch correction (Figure S1C–D). The results of both the distribution boxplots and PCA plots indicated that batch effects were substantially mitigated across the samples in the READ dataset.

### 3.2 DEGs related to drug resistance and mitophagy in rectal cancer

**Figure 2:**
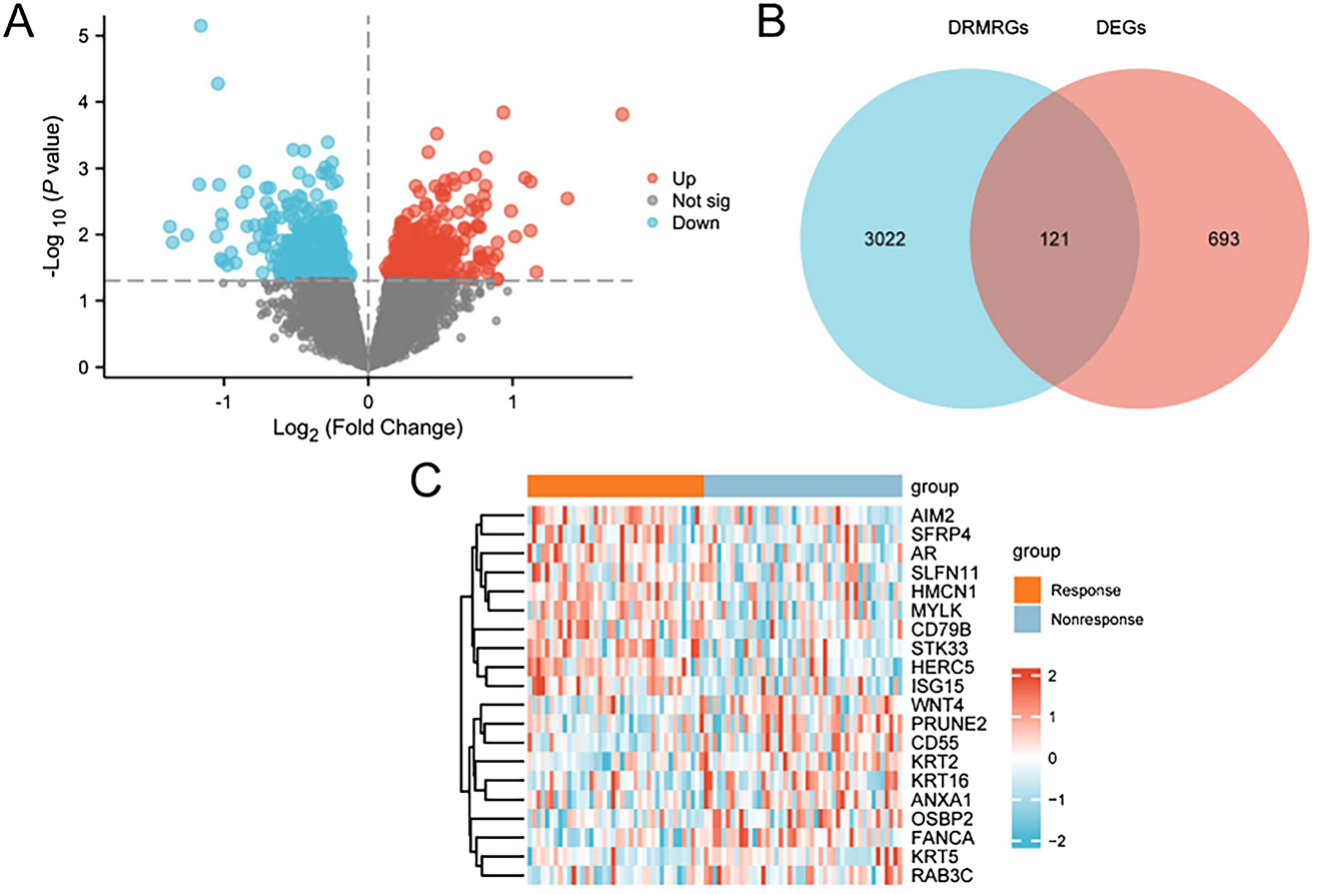
Differential gene expression analysis. A. Volcano plot of DEGs between the radiotherapy-responsive and non-responsive groups in the combined GEO datasets. B. Venn diagram showing overlap between DEGs and DRMRGs, identifying the subset of DRMRDEGs. C. Heatmap of the top 10 upregulated and top 10 downregulated DRMRDEGs ranked by logFC. In the heatmap: Blue indicates control samples; orange indicates READ samples. Expression values are color-coded from low (blue) to high (red).

As described previously, the combined datasets were categorized into radiotherapy-responsive and non-responsive groups. To examine gene expression differences between the two groups, differential expression analysis was conducted using the R package limma. A total of 814 DEGs met the criteria of |logFC| > 0 and *p*-value < 0.05. Among these, 372 genes were upregulated (logFC > 0 and *p* < 0.05), while 442 genes were downregulated (logFC < 0 and *p* < 0.05), as shown in the volcano plot (Figure 2A).

To identify DRMRDEGs, the 814 DEGs were intersected with the previously curated set of DRMRGs. This intersection yielded 121 DRMRDEGs, as illustrated in the Venn diagram (Figure 2B). Expression profiles of these genes, including the top 10 upregulated and top 10 downregulated DRMRDEGs ranked by logFC, were further analyzed and visualized in the heatmap (Figure 2C).

### 3.3 Differential expression verification and ROC curve analysis

**Figure 3:**
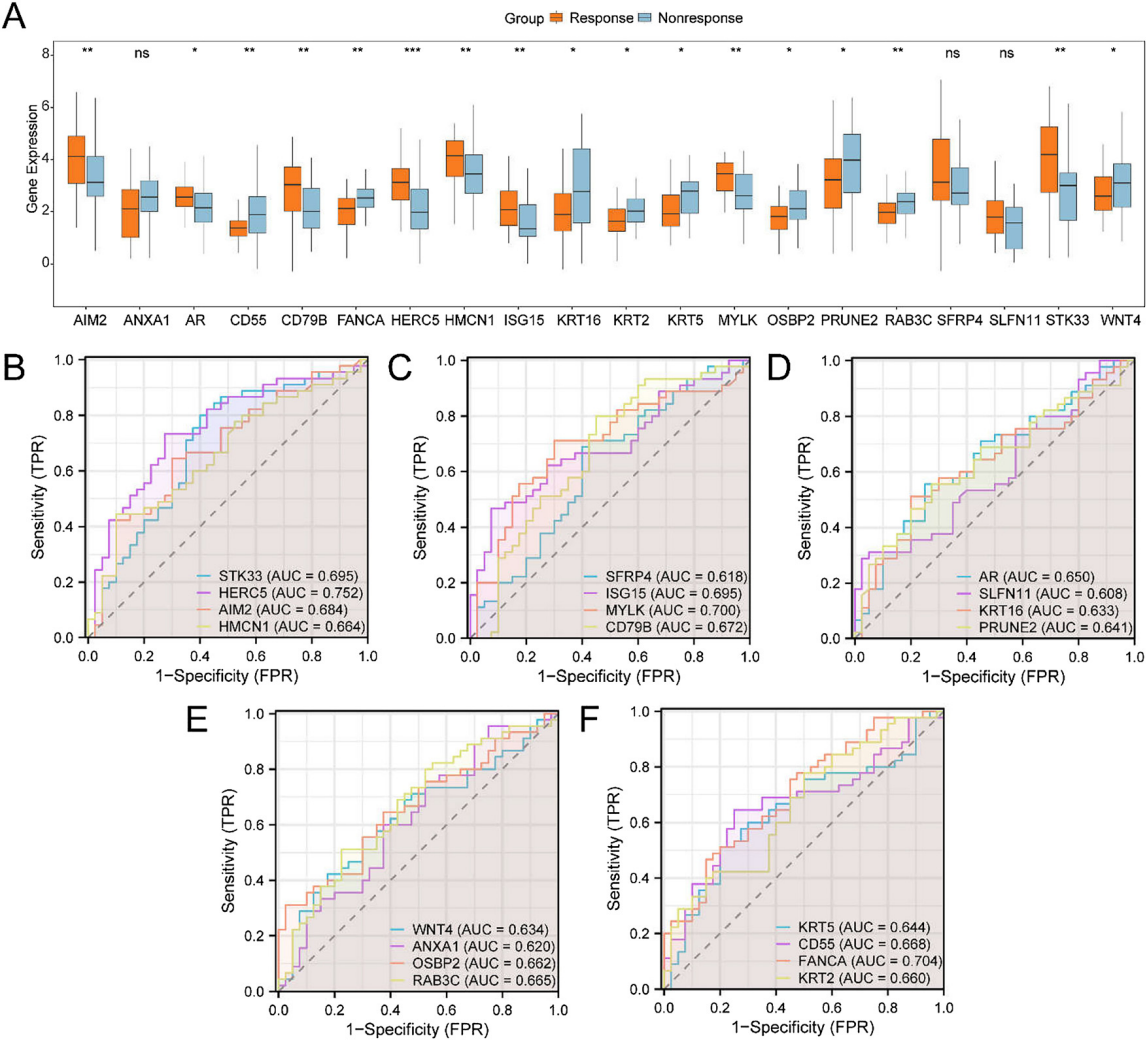
Differential expression validation and ROC curve analysis. A. Group comparison plot of the top 10 upregulated and top 10 downregulated DRMRDEGs (ranked by logFC) between the radiotherapy-responsive and non-responsive groups in the combined GEO datasets. B–F. ROC curves evaluating the diagnostic performance of selected DRMRDEGs in distinguishing between radiotherapy-responsive and non-responsive groups. B. ROC curves for *STK33, HERC5, AIM2,* and *HMCN1*. C. ROC curves for *SFRP4, ISG15, MYLK,* and *CD79B*. D. ROC curves for *AR, SLFN11, KRT16,* and *PRUNE2*. E. ROC curves for *WNT4, ANXA1, OSBP2,* and *RAB3C*. F. ROC curves for *KRT5, CD55, FANCA,* and *KRT2*. AUC, area under the curve; TPR, true positive rate; FPR, false positive rate. Statistical significance is annotated as follows: ns indicates *p* ≥ 0.05 (not significant); * represents *p* < 0.05 (significant); ** indicates *p* < 0.01 (highly significant); *** indicates *p* < 0.001 (very highly significant). An AUC > 0.5 suggests that gene expression is positively associated with the event (e.g., radiotherapy response), with values approaching 1.0 indicating better diagnostic performance. AUC values between 0.5 and 0.7 reflect low diagnostic accuracy, while values between 0.7 and 0.9 indicate moderate accuracy. In the group comparison plots, orange represents the radiotherapy-responsive group, and blue represents the non-responsive group.

To assess expression differences of DRMRDEGs in the combined datasets, the top 10 upregulated and top 10 downregulated DRMRDEGs, ranked by logFC, were analyzed between the radiotherapy-responsive and non-responsive groups. The results were visualized using group comparison plots (Figure 3A).

Differential expression analysis identified several DRMRDEGs prominently associated with drug resistance and mitophagy in rectal adenocarcinoma, including *AIM2*, *CD55*, *CD79B*, Fanconi anemia complementation group A (*FANCA*), *HMCN1*, *ISG15*, and *MYLK*. Of these, seven DRMRDEGs – *AR*, *KRT16*, *KRT2*, *KRT5*, *OSBP2*, *PRUNE2*, and *WNT4* – showed statistically significant differential expression (*p* < 0.05). In addition, *RAB3C* and *STK33* exhibited significant differences (*p* < 0.01), while *HERC5* demonstrated highly significant differential expression (*p* < 0.001), suggesting a potential role in drug resistance and mitophagy-related pathways.

ROC curve analysis (Figure 3B–F) was performed to evaluate the diagnostic performance of selected DRMRDEGs in differentiating between the radiotherapy-responsive and non-responsive groups. Among these, *HERC5*, *MYLK*, and *FANCA* exhibited moderate diagnostic accuracy, with AUC values ranging from 0.7 to 0.9. A total of 17 DRMRDEGs – including *HMCN1*, *SFRP4*, *ISG15*, *CD79B*, *AR*, *SLFN11*, *KRT16*, *PRUNE2*, *KRT5*, *CD55*, *KRT2*, *WNT4*, and *ANXA1* – were identified as significantly associated with drug resistance and mitophagy mechanisms. In contrast, *OSBP2* and *RAB3C* demonstrated lower diagnostic performance (0.5 < AUC < 0.7), indicating limited utility as predictive biomarkers in this context.

### 3.4 Construction of prognostic risk model for rectal cancer

To construct the prognostic risk model for READ, LASSO regression analysis was conducted using the 121 identified DRMRDEGs. The resulting LASSO regression model is presented in Figure S2A, with the variable coefficient trajectories depicted in Figure S2B. A total of 15 genes were retained as prognostic model genes based on the optimal lambda value.

The RiskScore for each sample was calculated using the following formula:

$$\text{RiskScore}=C1\text{orf}35\;*\;\left(0.175\right)+\text{PDGFRA }*\;\left(‐0.195\right)+\text{AATF }*\;\left(‐0.297\right)$$

$$+\text{FANCA }*\;\left(‐0.373\right)+\text{CRBN }*\;\left(‐0.372\right)+\text{LAMP}2\;*\;\left(0.252\right)+\text{RAB}3C+$$

$$\text{HLA }‐\;C\;*\;*\;\left(2.91\right)\;\left(0.302\right)+\text{EIF}3L\text{ AIP }*\;*\;\left(0.015\right)+\left(1.09\right)+\left(0.48\right)+$$

$$\text{WNT}4\text{ PES}1\;*\;*\;\left(0.399\right)+\mathrm{SEC}13\;*\;\left(‐0.03\right)+$$

$$\text{MAP}3K6\;*\;\left(0.556\right)+\text{OXA}1L\;*\;\left(0.168\right)$$

### 3.5 Prognostic analysis of prognostic risk model for rectal cancer

**Figure 4:**
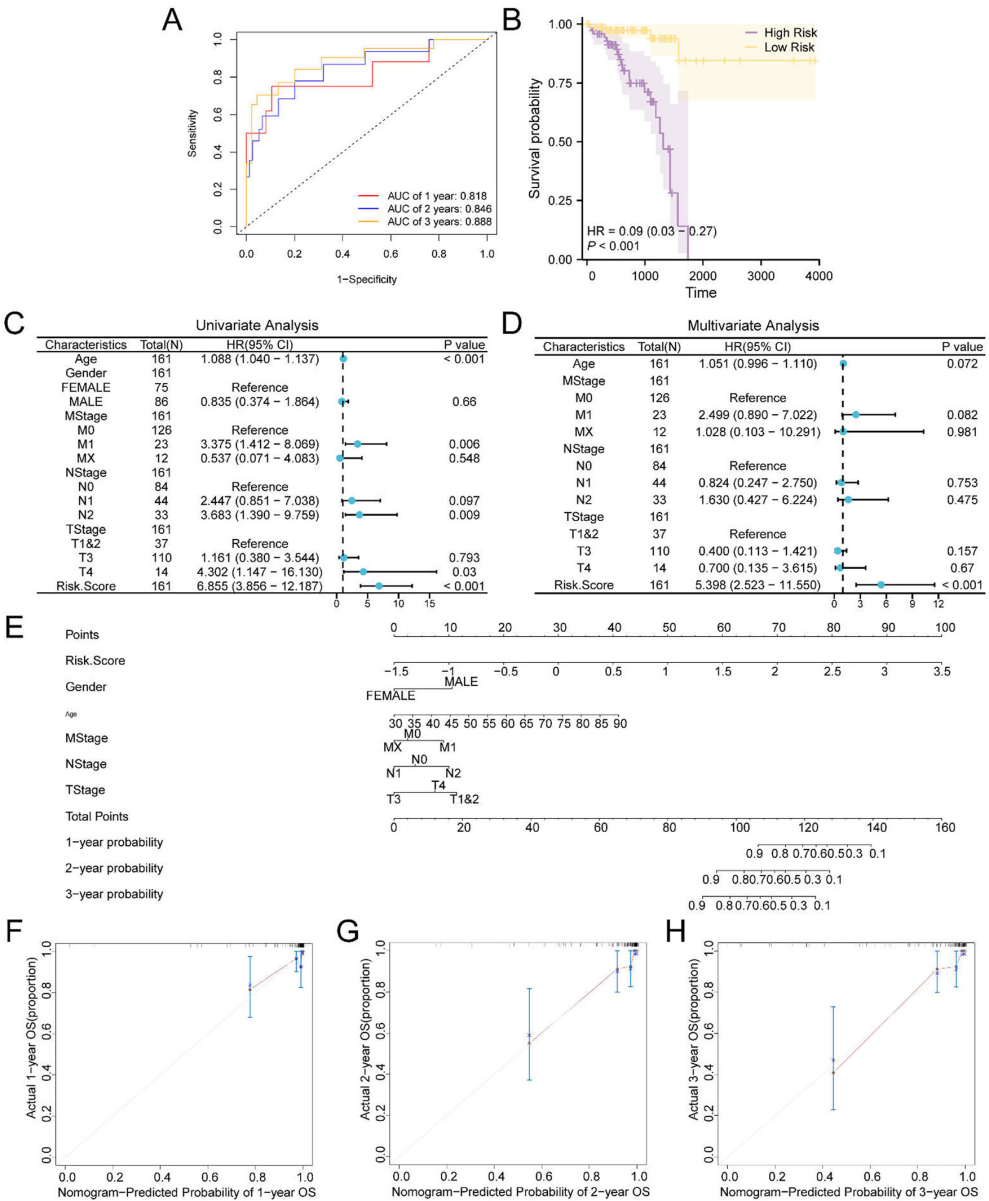
Prognostic analysis. A. Time-dependent ROC curves predicting 1-, 2-, and 3-year overall survival (OS) in READ samples from the TCGA-READ cohort. B. Kaplan–Meier (KM) survival curves comparing OS between high- and low-risk groups based on the median RiskScore. C–D. Forest plots showing the results of univariate (C) and multivariate (D) cox regression analyses of RiskScore and clinical variables. E. Nomogram integrating RiskScore and clinical variables to estimate 1-, 2-, and 3-year survival probabilities. F–H. Calibration curves assessing the predictive performance of the nomogram at 1 year (F), 2 years (G), and 3 years (H), comparing predicted survival probabilities with observed outcomes. An AUC > 0.5 indicates a positive association between the molecular signature and the survival outcome; values between 0.7 and 0.9 denote moderate predictive accuracy. A *p*-value < 0.001 was considered highly statistically significant.

As depicted in the time-dependent ROC curve (Figure 4A), the prognostic risk model for READ demonstrated moderate predictive accuracy for 1-, 2-, and 3-year OS, with AUC values ranging between 0.7 and 0.9. Additionally, KM survival analysis (Figure 4B) indicated a statistically significant difference in OS between the high- and low-risk groups, stratified by the median RiskScore (*p* < 0.001).

As described previously, univariate Cox regression analysis was conducted based on the RiskScore group (dichotomized by the median) in combination with OS and associated clinical variables of the TCGA-READ samples. Variables with *p*-values < 0.10 were retained, and the results were visualized using forest plots (Figure 4C and D), with additional details provided in Table S3. Among the included variables, the RiskScore remained statistically significant (*p* < 0.05), supporting its role as an independent prognostic factor.

To further evaluate the clinical utility of the prognostic model, a nomogram was constructed (Figure 4E) based on the results of the Cox regression analyses. This nomogram incorporated the RiskScore along with six clinical variables from the READ cohort. The RiskScore contributed the highest relative prognostic weight, while the M stage exhibited the lowest.

Calibration curves were subsequently generated to assess the model’s predictive performance for 1-, 2-, and 3-year OS (Figure 4F–H). In these plots, the *x*-axis represents predicted survival probability, and the *y*-axis represents observed survival probability. The degree of alignment between the plotted curve and the diagonal reference line reflects the model’s calibration accuracy. Among the time points evaluated, the 3-year calibration curve demonstrated the closest alignment with the ideal reference, suggesting superior predictive accuracy at this time point.

### 3.6 Differential expression verification and ROC curve analysis between high- and low-risk groups

**Figure 5:**
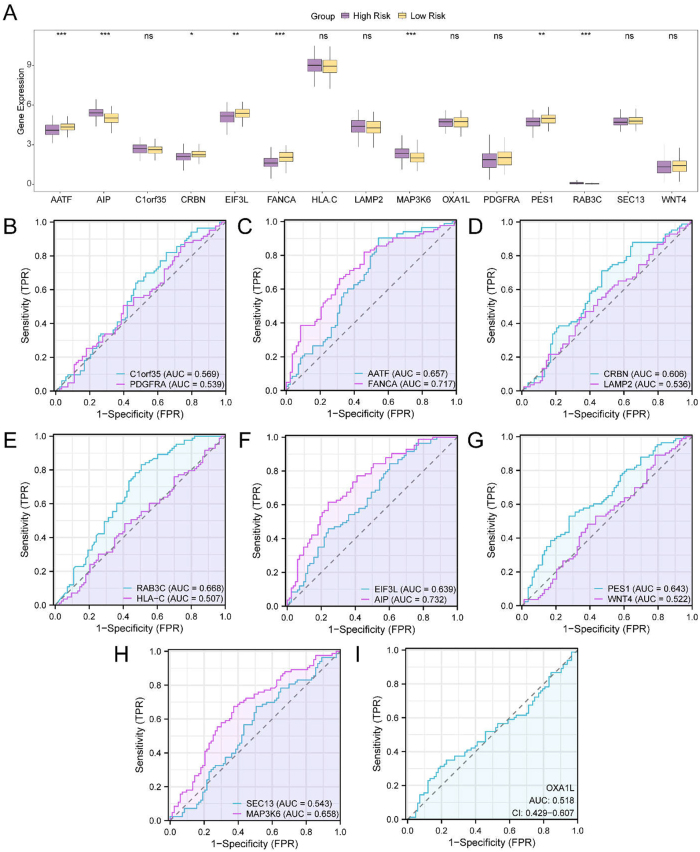
Differential expression validation and ROC curve analysis of model genes. A. Group comparison of model gene expression between high- and low-risk groups in READ samples from the TCGA-READ dataset. B–I. Receiver operating characteristic (ROC) curve analyses of the 15 model genes: B. *C1orf35* and *PDGFRA*. C. *AATF* and *FANCA*. D. *CRBN* and *LAMP2*. E. *RAB3C* and *HLA-C*. F. *EIF3L* and *AIP*. G. *PES1* and *WNT4*. H. *SEC13* and *MAP3K6*. I. *OXA1L*. Statistical significance is indicated as follows: ns = not significant (*p* ≥ 0.05); * = *p* < 0.05; ** = *p* < 0.01; *** = *p* < 0.001. An AUC > 0.5 indicates a positive predictive trend; values between 0.5 and 0.7 reflect low diagnostic accuracy, while values between 0.7 and 0.9 indicate moderate accuracy. In the group comparison plot, purple denotes the high-risk group and yellow denotes the low-risk group.

READ samples were stratified into high- and low-risk groups based on the median RiskScore derived from the prognostic model. Differential expression analysis was then performed for the 15 model-selected genes, comparing expression levels between the two risk groups. The results are illustrated in a group comparison plot (Figure 5A).

Among the model genes, *CRBN* showed statistically significant differential expression between high- and low-risk groups (*p* < 0.05). *EIF3L* and *PES1* exhibited highly significant differences (*p* < 0.01), while five genes – apoptosis antagonizing transcription factor (*AATF*), *AIP*, *FANCA*, *MAP3K6*, and *RAB3C* – demonstrated strongly significant expression differences (*p* < 0.001), suggesting their potential relevance in risk stratification for READ.

ROC curve analysis was conducted to assess the predictive performance of individual model genes in distinguishing between high- and low-risk groups (Figure 5B–I). Among the 15 genes, *FANCA* and *AIP* demonstrated moderate diagnostic accuracy, with AUC values ranging from 0.7 to 0.9. The remaining 13 genes – including *C1orf35*, platelet-derived growth factor receptor alpha (*PDGFRA*), *AATF*, *CRBN*, *LAMP2*, *RAB3C*, *HLA-C*, *EIF3L*, *PES1*, *WNT4*, *SEC13*, *MAP3K6*, and *OXA1L* – showed AUC values between 0.5 and 0.7, indicating relatively lower discriminatory performance for risk group classification.

### 3.7 Analytical results of SMs and CNVs

SMs within the 3,143 DRMRGs were analyzed in READ samples from the TCGA-READ dataset. The mutation spectrum is presented in Figure S3A. A total of nine major types of somatic mutations were identified, with missense mutations being the most frequent. Among the variant classifications, SNPs were the most prevalent, and cytosine-to-thymine transitions represented the most common single nucleotide variant.

The somatic mutation profiles of the 15 genes included in the prognostic model were further examined and ranked by mutation frequency. As shown in Figure S3B, *PDGFRA* and *RAB3C* exhibited the highest mutation rates, each detected in approximately 3 % of the READ samples.

CNVs in the 15 model genes were assessed using GISTIC2.0. CNVs were observed in 14 of the 15 genes (Figure S3C–D), including *OXA1L*, *MAP3K6*, *WNT4*, *EIF3L*, *RAB3C*, *PES1*, *PDGFRA*, *AIP*, *AATF*, *CRBN*, *SEC13*, *HLA-C*, *C1orf35*, and *LAMP2*.

### 3.8 Analytical results of GO and KEGG pathway enrichment

GO and KEGG pathway enrichment were performed to investigate the functional associations of the 121 DRMRDEGs in READ. Detailed enrichment results are provided in Table S4.

GO enrichment analysis revealed that the DRMRDEGs were predominantly involved in BP such as actin cytoskeleton reorganization, regulation of response to biotic stimulus, response to virus, defense response to symbiont, and defense response to virus. Enrichment in the CC category included cell-substrate junctions, focal adhesions, autophagosomes, phagocytic vesicles, and ficolin-1-rich granules.

**Figure 6:**
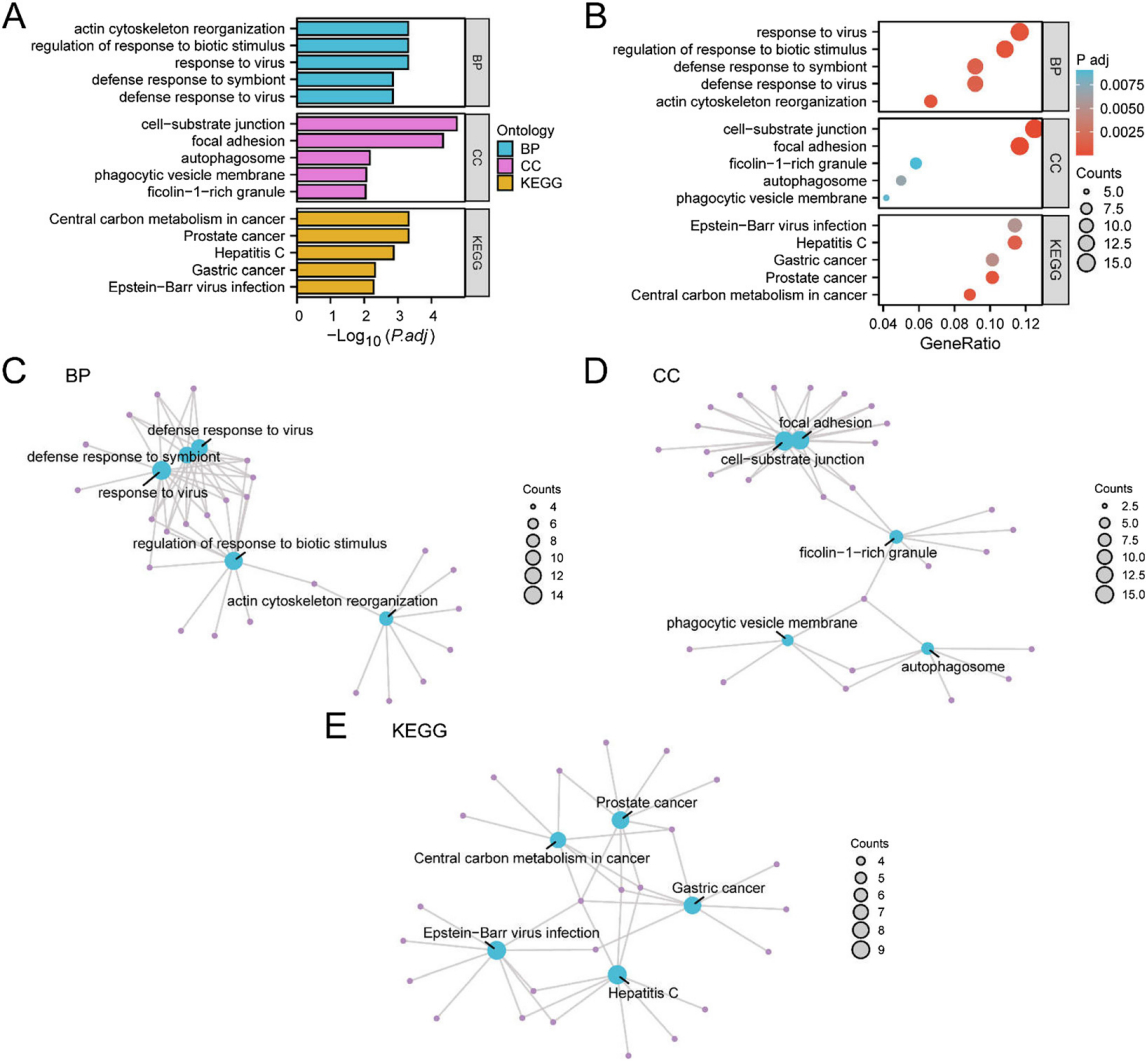
GO and KEGG enrichment analysis of DRMRDEGs. A–B. GO and KEGG enrichment analysis results for DRMRDEGs, visualized as a bar plot (A) and bubble plot (B). Enriched GO categories are classified into biological process (BP), cellular component (CC), and molecular function (MF); enriched KEGG pathways are also shown. GO and KEGG terms are presented on the *y*-axis. C–E. Network diagrams illustrating enrichment results for GO biological processes (C), GO cellular components (D), and KEGG pathways (E). Purple nodes represent enrichment terms; blue nodes represent associated genes; edges indicate gene–term associations. In the bubble plot, bubble size reflects the number of genes involved, and color represents the adjusted *p*-value (adj. *p*); red indicates smaller adj. *p*-values, while blue indicates larger adj. *p*-values. Enrichment significance was defined as adj. *p* < 0.05 and FDR (*q*-value) < 0.25. Multiple testing correction was performed using the benjamini–hochberg (BH) method.

KEGG pathway analysis identified significant enrichment in several pathways, including central carbon metabolism in cancer, prostate cancer, hepatitis C, gastric cancer, and Epstein–Barr virus infection. These enrichment findings were visualized using bar plots (Figure 6A) and bubble plots (Figure 6B).

To further illustrate the functional associations, network diagrams were generated to depict the relationships among enriched GO biological processes, cellular components, and KEGG pathways (Figure 6C–E). In these diagrams, nodes represent individual functional terms or pathways, and edges indicate shared gene annotations. Node size reflects the number of associated genes, with larger nodes indicating higher levels of gene enrichment. Notably, gene enrichment was most prominent at the cell-substrate junction level within the CC category.

### 3.9 Results of GSEA

GSEA was performed to evaluate the influence of gene expression levels on radiotherapy-related BPs in READ using the combined datasets. The analysis also explored the associations between affected CCs and MFs, as depicted in Figure S4A. Detailed results are provided in Table S5.

The GSEA results revealed significant associations between gene expression and multiple biologically relevant signaling pathways. Notably, pathways such as the interleukin-6 (IL-6) signaling pathway (Figure S4B), phosphoinositide 3-kinase (PI3K) signaling pathway (Figure S4C), Janus kinase–signal transducer and activator of transcription (JAK–STAT) signaling pathway (Figure S4D), and the PI3K–Akt signaling pathway (Figure S4E) were significantly enriched. These pathways may play key regulatory roles in modulating the radiotherapy response in READ.

### 3.10 Results of GSVA

GSVA was conducted on all genes from the combined datasets to assess pathway-level enrichment differences between the radiotherapy-responsive and non-responsive groups. The analysis utilized the gene set file c2. all.v2023.2. hs.symbols.gmt, with detailed results provided in Table S6. The top 10 positively and top 10 negatively enriched pathways (based on *p* < 0.05 and ranked by logFC) are displayed in a group comparison plot (Figure S5A).

All 20 pathways exhibited statistically significant enrichment differences between the radiotherapy-responsive and non-responsive groups (*p* < 0.05). Among these, seven pathways – EGF–EGFR–RAS–PI3K signaling, negative regulation of TCF-dependent signaling by DVL-interacting proteins, angiogenic targets of VHL–HIF2A upregulation, cerebral organic acidurias including diseases, ubiquinol biosynthesis, mitochondrial fatty acid synthesis, and the BARD1 pathway – showed differential enrichment across both groups.

Eleven additional pathways demonstrated higher statistical significance (*p* < 0.01), including TERT targets upregulation, interferon alpha response, DNAJB4 targets up, variant-amplified REL involvement in transcription, EGF–EGFR–PI3K signaling, variant EGF overexpression to PI3K signaling, amplification hotspot 29, IGH–MMSET fusion-driven transcriptional activation, familial hyperlipidemia type 5, and pathways associated with vitamin metabolism and poor lung cancer prognosis.

Two pathways – HGF targets induced by AKT1 (6 h) and familial hyperlipidemia type 1 – exhibited highly significant enrichment (*p* < 0.001) in both groups.

These pathway activity differences between the radiotherapy-responsive and non-responsive groups were further visualized in a heatmap (Figure S5B), highlighting the distinct enrichment patterns identified through GSVA.

### 3.11 Immune infiltration analysis results

The infiltration abundance of 22 immune cell types was estimated in the combined datasets using the CIBERSORT algorithm. The relative proportions of these immune cells across samples are presented in a bar chart (Figure S6A). The correlation matrix for the infiltration levels of the 22 immune cell types was visualized and presented as a heatmap (Figure S6B). Among the observed associations, the strongest positive correlation was identified between activated mast cells and resting natural killer (NK) cells (*r* = 0.43), while the strongest negative correlation was found between activated mast cells and resting mast cells (*r* = −0.73).

Spearman correlation analysis was conducted to evaluate associations between the expression levels of the prognostic model genes and the abundance of immune cell infiltration. The results were visualized using bubble plots (Figure S6C). Notably, *FANCA* showed the strongest positive correlation with activated mast cell infiltration (*r* = 0.50, *p* < 0.05) and the most pronounced negative correlation with resting mast cell infiltration (*r* = −0.491, *p* < 0.05).

### 3.12 Analytical results of immune infiltration in high- and low-risk groups

**Figure 7:**
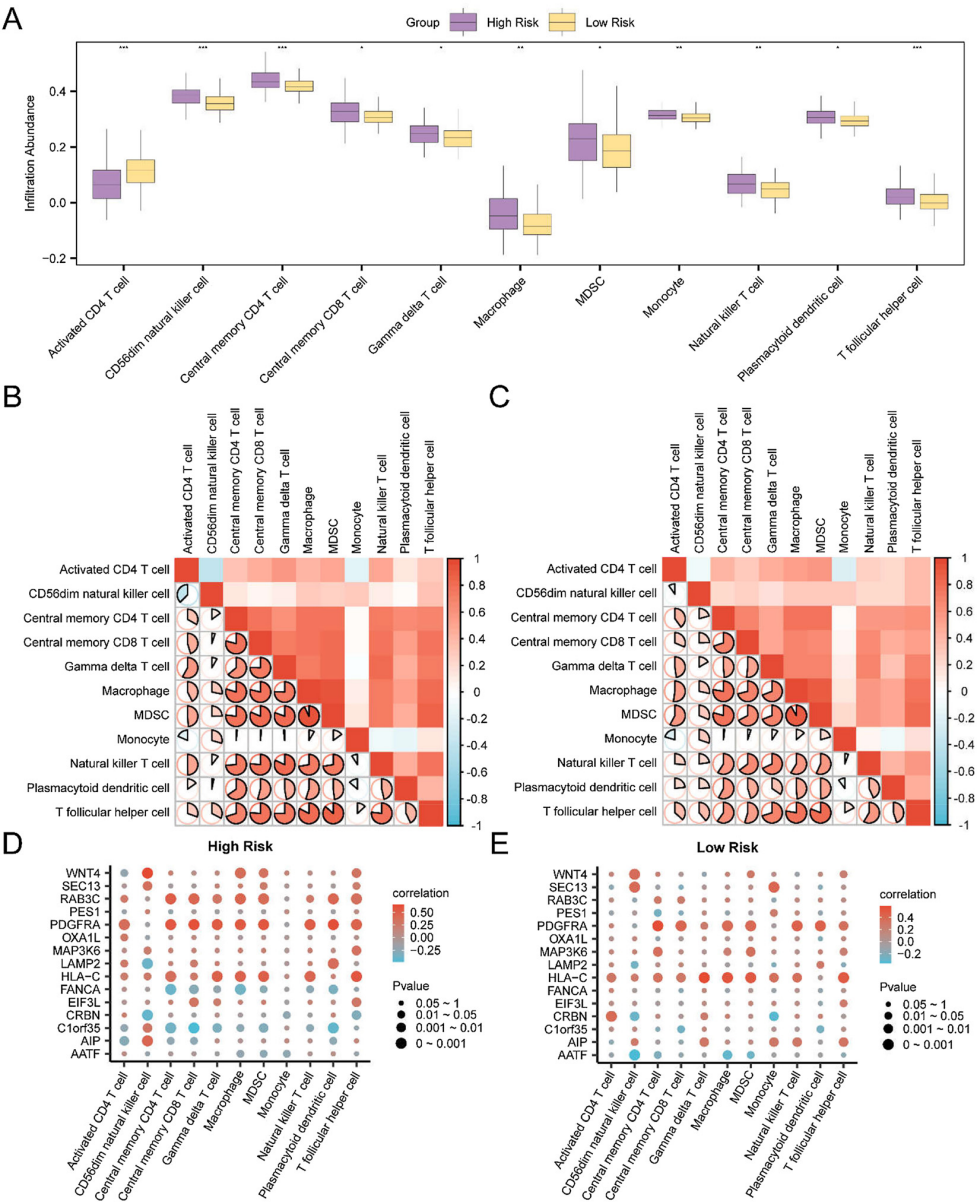
Immune infiltration analysis between risk groups based on the ssGSEA algorithm. A. Bubble plot showing the differences in immune cell infiltration between high- and low-risk groups in READ samples, based on ssGSEA. B–E. Enrichment plots from GSEA of the combined GEO datasets showing significant enrichment in key pathways: B. Interleukin-6 (IL6) signaling pathway. C. PI3KCI pathway. D. JAK–STAT signaling pathway. E. PI3K–AKT signaling pathway. In the bubble plot, bubble size corresponds to the number of enriched genes, while bubble color represents the NES value – red indicates higher NES values and blue indicates lower values. Significance thresholds were set at adj. *p* < 0.05 and FDR (*q*-value) < 0.25. Multiple testing correction was performed using the benjamini–hochberg (BH) method.

Immune infiltration abundance for 28 immune cell types was estimated in high- and low-risk groups using the ssGSEA algorithm, based on the gene expression matrix from TCGA-READ samples. Differences in immune cell infiltration between the two groups were visualized using a group comparison plot (Figure 7A).

Four immune cell types – central memory CD8^+^ T cells, γδ T cells, myeloid-derived suppressor cells, and plasmacytoid dendritic cells (pDCs) – exhibited statistically significant differences between the high- and low-risk groups according to the analysis (*p* < 0.05). Three immune cell types – macrophages, monocytes, and natural killer T (NKT) cells – showed highly significant differences (*p* < 0.01). Additionally, four immune cell types – activated CD4^+^ T cells, CD56^dim^ natural killer (NK) cells, central memory CD4^+^ T cells, and T follicular helper cells – demonstrated very highly significant differences (*p* < 0.001).

Correlation heatmaps depicting interrelationships among the infiltration levels of 11 selected immune cell types within each risk group are shown in Figure 7B and C. Most immune cell types exhibited strong positive correlations within both the high- and low-risk groups.

Further analysis was performed to assess the correlations between model gene expression and immune cell infiltration abundance, with results visualized using bubble plots (Figure 7D and E). In the high-risk group, *RAB3C*, *PDGFRA*, and *HLA-C* showed significant positive correlations with the majority of immune cell types (*p* < 0.05, *r* > 0), whereas *FANCA* and *C1orf35* were negatively correlated with immune infiltration (*p* < 0.05, *r* < 0). In the low-risk group, *PDGFRA* and *HLA-C* continued to exhibit significant positive correlations with most immune cell subsets (*p* < 0.05, *r* > 0).

### 3.13 IPS analysis

Comparative analysis of IPS classifications between the high- and low-risk groups in READ samples revealed statistically significant differences, as illustrated in Figure S7. The overall IPS classification differed significantly between the two groups, with a highly significant difference observed (*p* < 0.01). Additionally, both the IPS–PD1 and IPS–CTLA4 subcategories showed statistically significant variation between the groups (*p* < 0.05), suggesting potential differences in immune checkpoint responsiveness associated with risk stratification.

### 3.14 PCR validation of gene differential expression and pathway-related molecules

**Figure 8:**
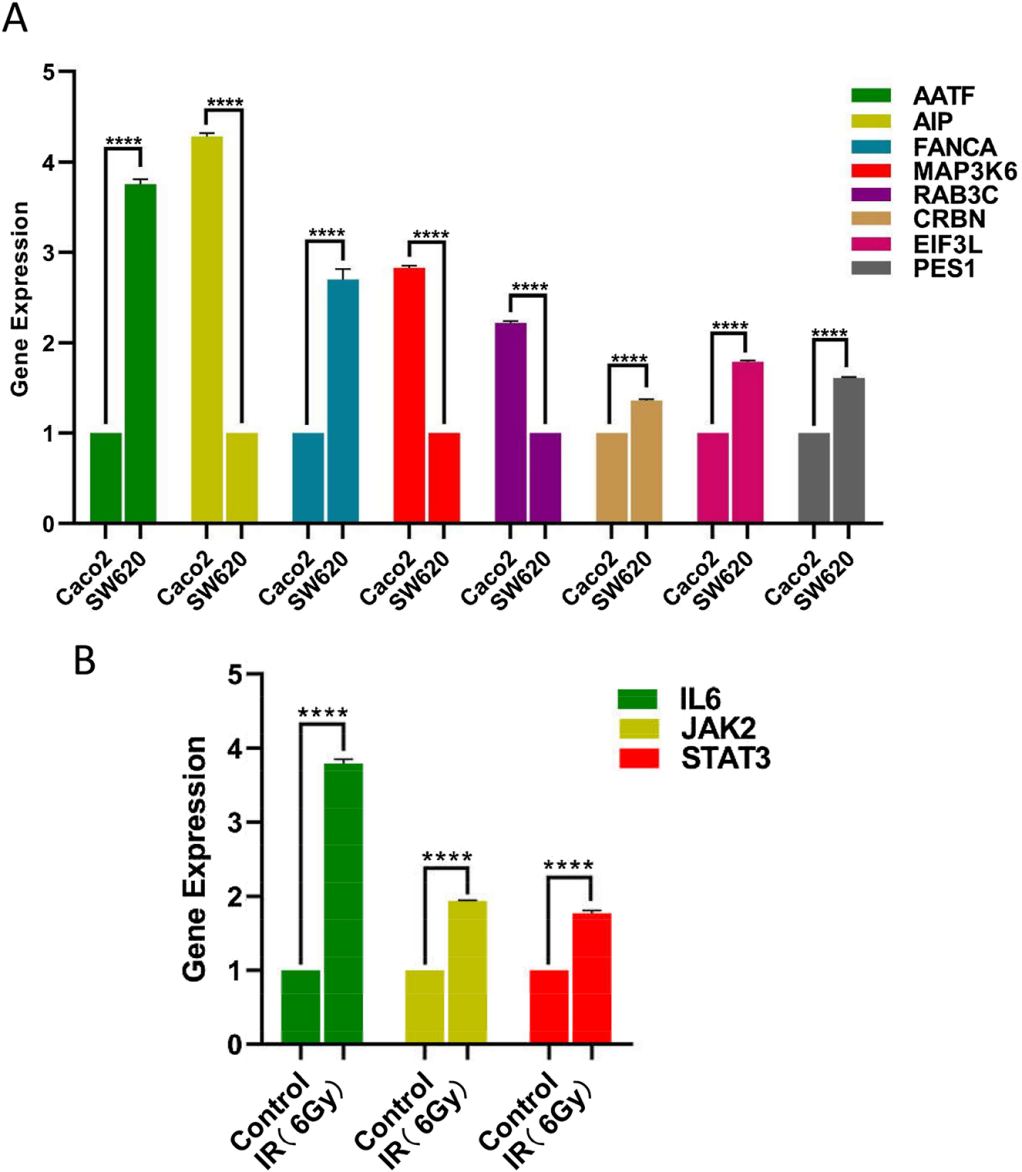
PCR validation of gene expression and pathway-associated molecules. A. Quantitative reverse transcription PCR (qRT-PCR) analysis of mRNA expression levels of *AATF*, *AIP*, *FANCA*, *MAP3K6*, *RAB3C*, *CRBN*, *EIF3L*, and *PES1* in SW620 (high-risk) and Caco2 (low-risk) cell lines. Statistical significance was determined using two-way ANOVA followed by Tukey’s multiple comparisons test. ****p < 0.0001. B. HCT116 cells were exposed to ionizing radiation (IR) or left untreated. After 48 h, qRT-PCR was used to quantify mRNA expression levels of *IL6*, *JAK2*, and *STAT3*. Statistical comparisons were performed using two-way ANOVA with Tukey’s post hoc test. ****p < 0.0001, compared with the control group.

RT–qPCR analysis revealed significant differences in mRNA expression levels of *AATF*, *AIP*, *FANCA*, *MAP3K6*, *RAB3C*, *CRBN*, *EIF3L*, and *PES1* between SW620 cells (representing the high-risk group) and Caco2 cells (representing the low-risk group), as shown in Figure 8A.

Additionally, transcript levels of *IL6*, *JAK2*, and *STAT3* were notably upregulated in irradiated HCT11 cells, as confirmed by real-time PCR analysis (Figure 8B).

## 4 Discussion

CRC is the third most commonly diagnosed malignancy worldwide and remains a major contributor to cancer-related morbidity and mortality. Its progression and metastatic potential are strongly associated with poor clinical outcomes and reduced overall survival, underscoring the limitations of current diagnostic and therapeutic strategies. The substantial heterogeneity observed in CRC presents a critical challenge in accurately predicting treatment response, thereby necessitating the development of more precise and individualized therapeutic approaches to improve patient outcomes and quality of life.

DRMRDEGs have recently been implicated in shaping the tumor microenvironment and influencing CRC cell behavior. Characterizing the interaction between these genes and the molecular features of the tumor may provide valuable insights into tumor progression and therapeutic resistance. In this context, DRMRDEGs represent promising candidates for the identification of novel prognostic biomarkers and potential therapeutic targets. The present study aimed to investigate the role of DRMRDEGs in CRC, with a focus on their expression patterns, clinical relevance, and functional implications in radiotherapy response.

Differential expression analysis, along with GO, KEGG pathway analysis, and GSEA, was conducted to identify key genes and signaling pathways associated with CRC prognosis and treatment response. The diagnostic potential of these genes was evaluated using ROC curve analysis. Furthermore, a prognostic risk model was constructed using LASSO regression to predict survival outcomes in patients with rectal adenocarcinoma. Immune cell infiltration patterns were characterized using the CIBERSORTx and ssGSEA algorithms, while SM and CNV analyses were conducted to explore the mutational landscape of the selected genes.

The findings of this study provide new insights into the molecular mechanisms underlying rectal cancer progression and therapeutic resistance, with implications for the development of novel diagnostic tools and targeted treatment strategies. Among the identified DRMRDEGs, *C1orf35*, *PDGFRA*, and *FANCA* were found to play prominent roles in modulating the tumor microenvironment, potentially influencing cancer cell proliferation, migration, and overall patient prognosis. Aberrant expression of these genes may result from epigenetic regulation, SM, or copy number alterations, suggesting the need for further mechanistic investigation to validate their clinical utility as biomarkers or therapeutic targets.

Through a comprehensive assessment of prognostic factors in rectal cancer, several genes, including *C1orf35*, *PDGFRA*, *FANCA*, and *AATF*, were found to be significantly associated with clinical outcomes. These genes participate in diverse cellular processes and have previously been implicated in tumorigenesis and cancer progression.

Although the precise biological function of *C1orf35* remains incompletely characterized, the present findings suggest a potential association between its expression and rectal cancer prognosis. Previous studies have reported an oncogenic role for *C1orf35* in multiple myeloma (MM), where it promotes cell cycle progression and supports cellular proliferation via regulation of *c-MYC* expression. These observations underscore the possible contribution of *C1orf35* to cancer progression and its potential relevance as a therapeutic target, particularly in tumors exhibiting dysregulated *c-MYC* activity [34].

*PDGFRA*, a key receptor in the PDGF signaling pathway, plays a critical role in regulating cellular proliferation, migration, and survival. In the present study, aberrant expression of *PDGFRA* was associated with poorer clinical outcomes in patients with rectal cancer. This finding is consistent with previous reports demonstrating that *PDGFRA* overexpression contributes to tumor aggressiveness and progression in multiple malignancies, including pancreatic and breast cancers [35], 36]. These results support the potential of *PDGFRA* as a therapeutic target in rectal cancer, particularly in patients with elevated expression profiles.

*FANCA*, a central component of the Fanconi anemia pathway, is essential for maintaining genomic integrity through its role in DNA interstrand crosslink repair. The current analysis revealed a significant association between altered *FANCA* expression and patient prognosis, likely reflecting its involvement in DNA damage response and repair processes. This observation is in line with prior evidence from a 2017 study indicating that *FANCA* loss of function contributes to increased genomic instability and tumorigenesis in various cancer types [3]. These findings highlight the relevance of *FANCA* as a potential prognostic biomarker and its importance in pathways that preserve genomic stability in rectal cancer.

*AATF* is a transcriptional regulator involved in the DNA damage response and cell cycle control. In this study, *AATF* expression was significantly associated with prognosis in rectal cancer, potentially due to its regulatory influence on genes involved in cell cycle progression and apoptosis. This observation is consistent with findings from a 2019 study, which demonstrated that *AATF* contributes to breast cancer progression through the modulation of cell cycle-related genes [37], further supporting its role in tumor development and progression.

Collectively, *C1orf35*, *PDGFRA*, *FANCA*, and *AATF* were identified as key molecular components in the landscape of rectal cancer, each demonstrating consistent associations with clinical outcomes. These genes not only serve as potential prognostic biomarkers but also represent promising candidates for targeted therapeutic intervention. The findings of this study offer novel insights into their functional roles in rectal cancer biology and provide a foundation for future translational and clinical applications.

GSEA revealed significant enrichment of several signaling pathways among genes from the combined datasets, including the IL6 signaling pathway, PI3K–CI pathway, JAK–STAT signaling pathway, and PI3K–AKT signaling pathway. These pathways are well-characterized in cancer biology and are known to regulate key cellular processes such as proliferation, survival, and therapeutic resistance, including resistance to radiotherapy in rectal cancer [38], [39], [40].

Specifically, the IL6 signaling pathway has been implicated in shaping the inflammatory tumor microenvironment and promoting the development of radioresistance. The PI3K–AKT pathway is a central regulator of cell survival and has been previously associated with modulating radiotherapy response. The enrichment of these pathways in the current analysis suggests their potential involvement in influencing treatment outcomes in rectal cancer, in concordance with prior findings.

Immune infiltration analysis using both the CIBERSORTx algorithm and ssGSEA revealed significant correlations between immune cell subsets and the expression of DRMRDEGs. Among these, *FANCA* exhibited a notable positive correlation with activated mast cells and a negative correlation with resting mast cells. These findings align with prior studies implicating *FANCA* in the DNA damage response and its emerging role in immune regulation. Activated mast cells within the tumor microenvironment have been associated with enhanced immune activation and, in some contexts, improved responsiveness to immunotherapy. The observed correlation suggests that *FANCA* may influence the immune landscape in rectal cancer, potentially modulating anti-tumor immunity. These insights highlight the potential of *FANCA* not only as a prognostic biomarker but also as a candidate for therapeutic strategies involving immune modulation.

The IPS analysis revealed statistically significant differences in IPS classifications between the high- and low-risk groups, suggesting potential variability in immunotherapy responsiveness. These findings are consistent with emerging evidence supporting the use of molecular and immunological biomarkers to predict response to immune checkpoint inhibitors. The identification of such biomarkers is critical for advancing personalized treatment strategies in rectal cancer, as it enables more accurate selection of patients who are most likely to benefit from immunotherapeutic interventions.

The RiskScore model developed in this study, based on 15 DRMRDEGs, demonstrates strong potential for clinical application in the context of rectal cancer radiotherapy. By quantifying molecular risk characteristics, this model enables precise stratification of patients according to their predicted radiotherapy sensitivity, thereby supporting informed clinical decision-making. Patients classified as low-risk by the model exhibited higher radiotherapy response rates, suggesting that standard radiotherapy regimens may be sufficient for this subgroup, potentially minimizing overtreatment-related toxicity. Conversely, high-risk patients, who may exhibit inherent radioresistance, could benefit from treatment intensification, including combination therapies such as chemotherapy (e.g., capecitabine), targeted therapies (e.g., anti-angiogenic agents), or immune checkpoint inhibitors (e.g., PD-1 inhibitors), aligning with current trends in individualized treatment strategies for rectal cancer [41], 42].

This model may be integrated into routine clinical workflows, particularly during preoperative neoadjuvant radiotherapy. RiskScore assessment could be used to predict radiotherapy response in advance, enabling early adaptation of treatment intensity to improve tumor downstaging and sphincter preservation rates [43]. Moreover, key genes within the model, such as *FANCA* and *PDGFRA*, not only serve as prognostic markers but also highlight molecular pathways (e.g., DNA repair, PI3K–AKT signaling) that represent actionable therapeutic targets. These findings lay the groundwork for an integrated “prediction–treatment–monitoring” framework, supporting precision medicine approaches in rectal cancer management [44], 45].

## 5 Study limitations and future directions

Despite the promising results, this study has several limitations that warrant consideration:

1. Lack of Functional Validation

While the RiskScore model was constructed using robust bioinformatic analyses and supported by clinical correlation data, functional validation through *in vitro* and *in vivo* experiments is lacking. Assays such as gene knockdown/overexpression, clonogenic survival under irradiation, and mechanistic pathway studies are needed to confirm the causal role of the identified DRMRDEGs in modulating radiotherapy sensitivity. These experiments are essential to strengthen the biological plausibility and mechanistic underpinnings of the model, as recommended in recent prognostic model validation frameworks.

2. Absence of External Validation in Independent Cohorts

Although internal validation was conducted using the TCGA-READ and combined GEO datasets, external validation in multi-center cohorts is required to confirm the model’s generalizability. Differences in patient demographics, treatment protocols, and follow-up standards could influence the model’s performance. Independent validation in diverse clinical populations will be critical to establish its robustness and applicability in real-world settings.

3. Lack of Clinical Stratification and Decision Impact Analysis

The model’s performance was not evaluated across clinical subgroups (e.g., by tumor stage, age, or prior treatment). Additionally, the study did not assess whether incorporating the RiskScore into clinical decision-making workflows would alter treatment strategies or improve patient outcomes. Future work should include subgroup analyses and prospective clinical utility studies to demonstrate the model’s practical value beyond prognostic prediction.

4. Unexplored Association with Radiotherapy-Related Toxicity

The relationship between RiskScore and radiotherapy-induced toxicity was not investigated. As radiotherapy efficacy and adverse effects are often interrelated, understanding whether high-risk patients are also more susceptible to treatment-related toxicities could inform further refinement of individualized treatment strategies. This area represents an important direction for future clinical research.

Despite these limitations, the study provides a comprehensive investigation into the molecular mechanisms underlying rectal cancer pathogenesis and introduces a robust prognostic scoring model based on DRMRDEGs. Further experimental and clinical validation is warranted to confirm the mechanistic roles and therapeutic relevance of the identified genes and pathways. Nonetheless, the findings offer a valuable foundation for future research aimed at elucidating molecular targets and facilitating the development of clinically actionable strategies for the personalized management of rectal cancer.

## Supplementary Material

Supplementary Material

Supplementary Material

Supplementary Material

Supplementary Material

Supplementary Material

Supplementary Material

Supplementary Material

Supplementary Material
