# Supplementary figures and images for: Integrated genomic and clinical modeling for prognostic assessment of radiotherapy response in rectal neoplasms
Source: Open Life Sci. 2025 Dec 30;20(1):20251199. doi: 10.1515/biol-2025-1199 (PMC13011606; doi:10.1515/biol-2025-1199)

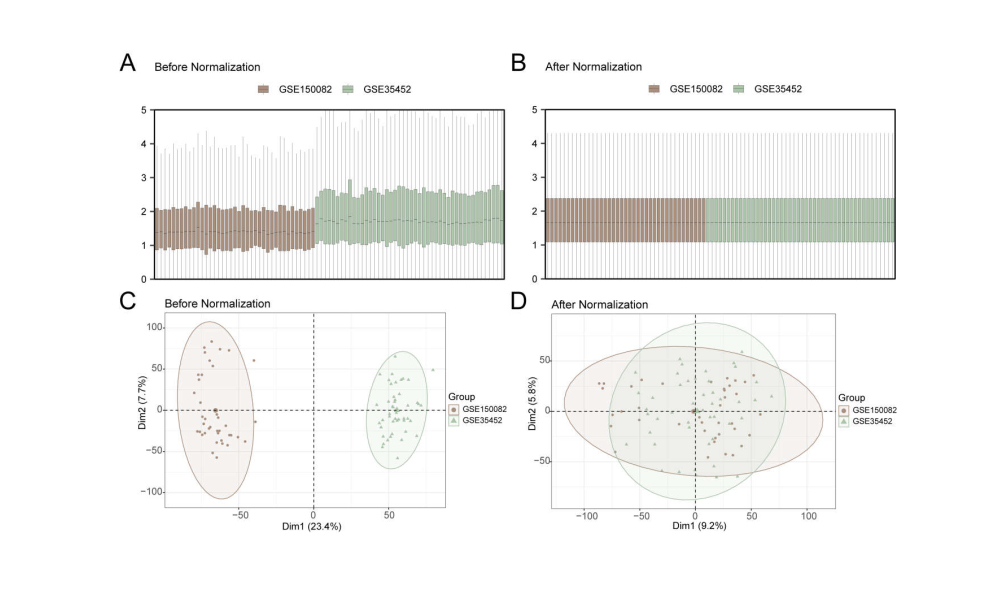

Supplement: Supplementary file 1 — Supplementary Material [file j_biol-2025-1199_suppl_001.jpg]

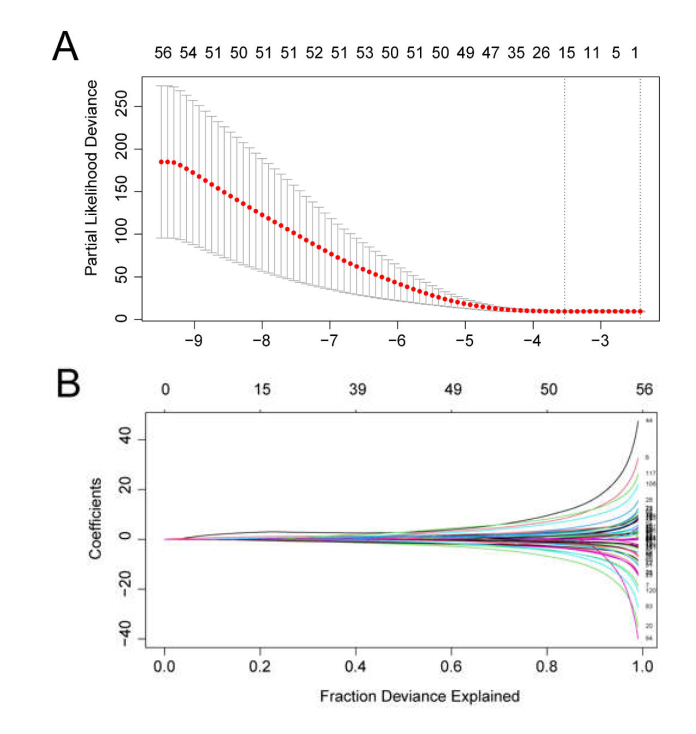

Supplement: Supplementary file 2 — Supplementary Material [file j_biol-2025-1199_suppl_002.jpg]

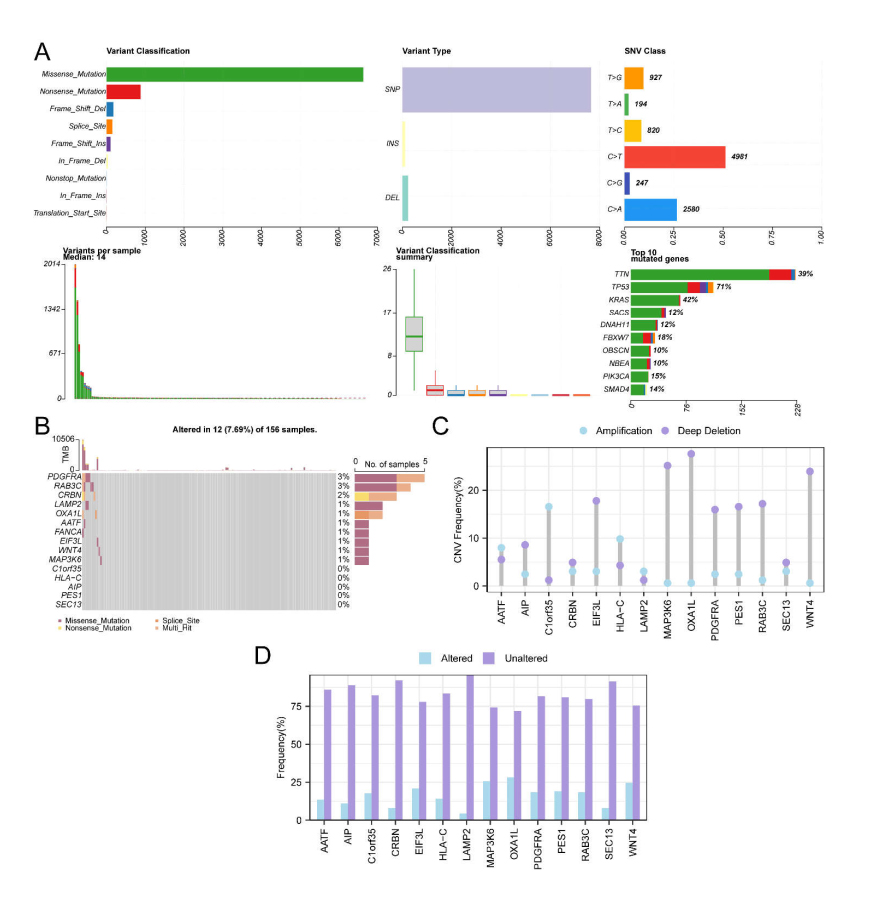

Supplement: Supplementary file 3 — Supplementary Material [file j_biol-2025-1199_suppl_003.jpg]

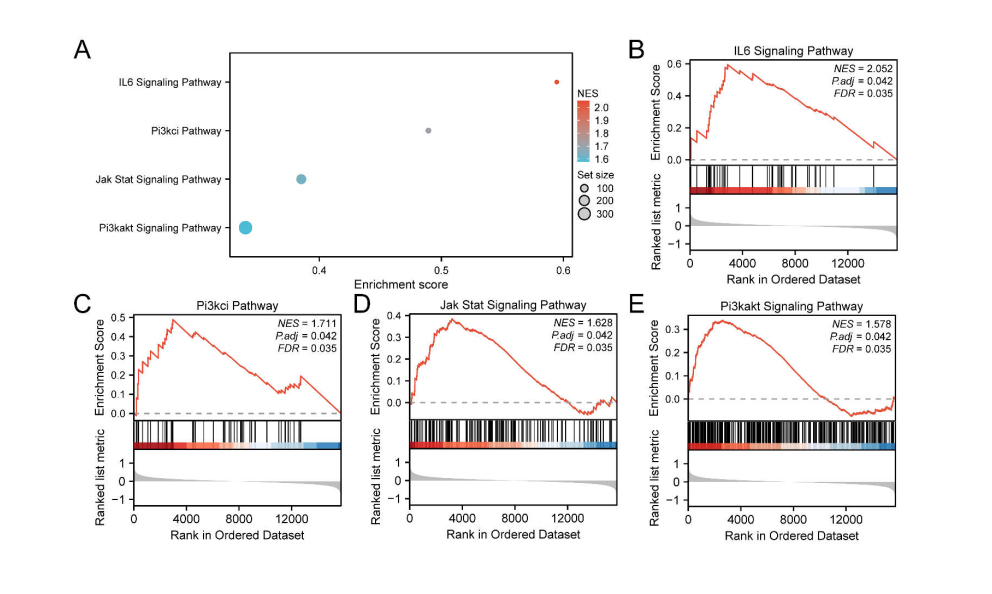

Supplement: Supplementary file 4 — Supplementary Material [file j_biol-2025-1199_suppl_004.jpg]

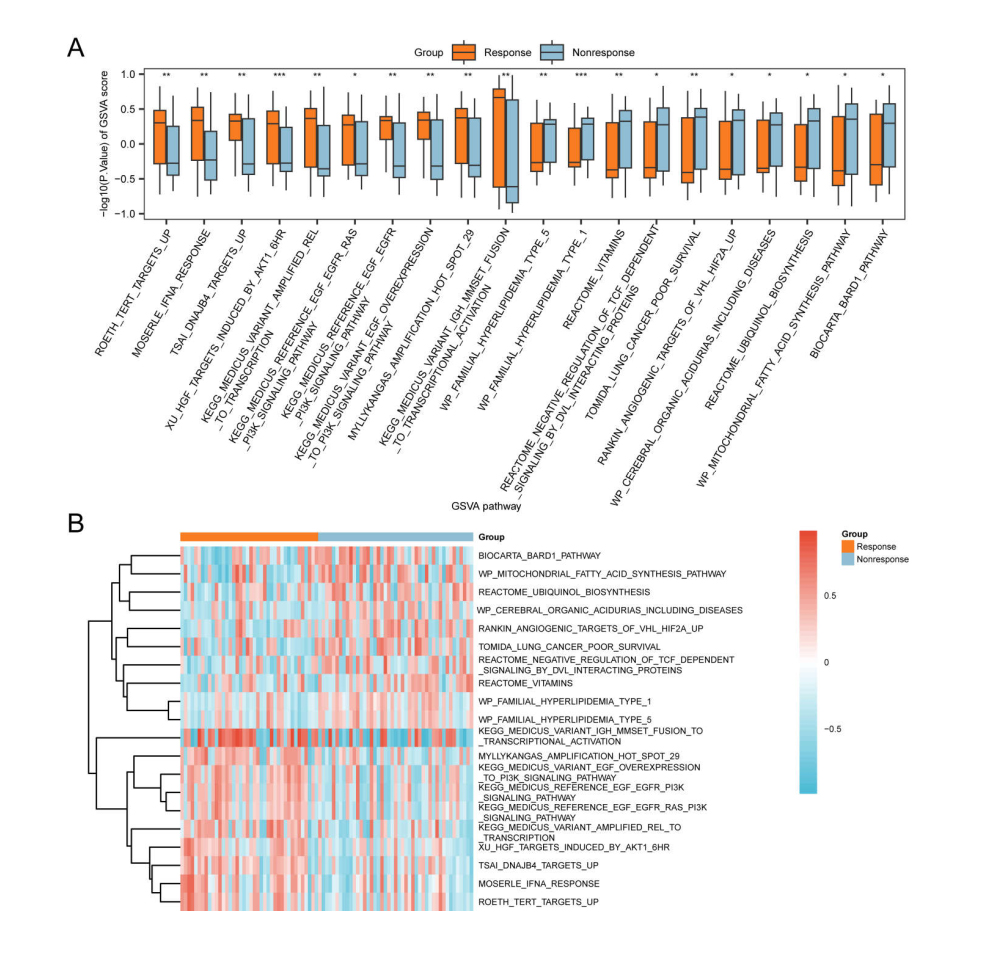

Supplement: Supplementary file 5 — Supplementary Material [file j_biol-2025-1199_suppl_005.jpg]

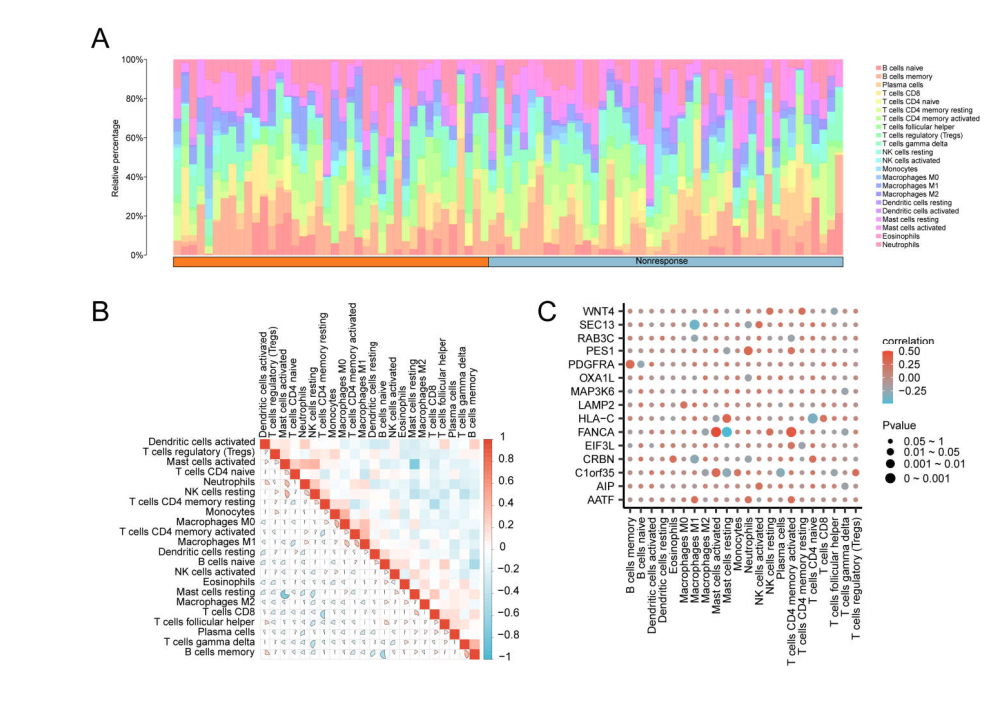

Supplement: Supplementary file 6 — Supplementary Material [file j_biol-2025-1199_suppl_006.jpg]

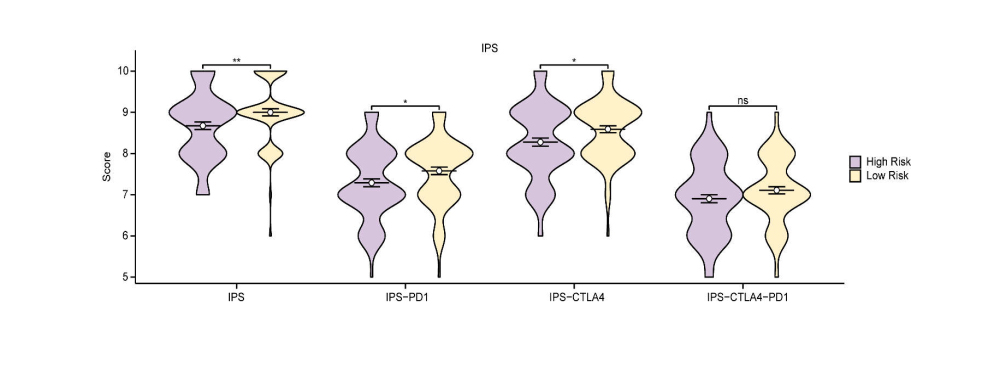

Supplement: Supplementary file 7 — Supplementary Material [file j_biol-2025-1199_suppl_007.jpg]
