# Supplementary material for: Integrated genomic and clinical modeling for prognostic assessment of radiotherapy response in rectal neoplasms
Source: Open Life Sci. 2025 Dec 30;20(1):20251199. doi: 10.1515/biol-2025-1199 (PMC13011606; doi:10.1515/biol-2025-1199)
Supplement: Supplementary file 8 — Supplementary Material [file j_biol-2025-1199_suppl_008.docx]

Fig.S1 Batch Effects Removal of GSE150082 and GSE35452

A. Box plot of Combined GEO Datasets distribution before batch removal. B. Post-batch integrated GEO Datasets (Combined Datasets) distribution boxplots. C. PCA plot of the integrated GEO Datasets (Combined Datasets) before debatching. D. PCA plot of GEO Datasets integrated after debatching (Combined Datasets). PCA, Principal Component Analysis; READ, Rectum Adenocarcinoma. The brown is the rectal cancer (READ) radiotherapy dataset GSE150082, and the green is the rectal cancer (READ) radiotherapy dataset GSE35452.

Fig.S2 LASSO Regression Analysis

A-B. Plots of prognostic risk models (A) and variable trajectories (B) of the LASSO regression model. LASSO, Least Absolute Shrinkage and Selection Operator.

Fig.S3 CNV and Somatic Mutation Analysis

A. Display of somatic mutations (SM) of drug resistance & mitophagy-related genes (DRMRGs) in rectal cancer (READ) samples from the rectal cancer dataset (TCGA-READ). B. Demonstration of somatic mutations (SM) of Model Genes in rectal cancer (READ) samples from the rectal cancer dataset (TCGA-READ). C-D. Copy number variation (CNV) of Model Genes in rectal cancer (READ) samples from the rectal cancer dataset (TCGA-READ) are shown. TCGA, The Cancer Genome Atlas; READ, Rectum Adenocarcinoma; DRMRGs, Drug Resistance&Mitophagy-Related Genes; SM, Somatic Mutation; CNV, Copy Number Variations; SNP, Single Nucleotide Polymorphism.

Fig.S4 GSEA for Combined Datasets

Fig.S5 GSVA for Combined Datasets

A-B. Group comparison plots (A) and heat maps (B) of gene set variation analysis (GSVA) results between Response and Nonresponse groups in the integrated GEO Datasets (Combined Datasets). TCGA, The Cancer Genome Atlas; READ, Rectum Adenocarcinoma; GSVA, Gene Set Variation Analysis. * represents p value < 0.05, statistically significant; ** represents p value < 0.01, highly statistically significant; *** represents p value < 0.001 and highly statistically significant. Orange represents the Response group, and blue represents the Nonresponse group. Blue represents low enrichment and red represents high enrichment in the heat map. The screening criterion for gene set variation analysis (GSVA) was a p-value < 0.05.

Fig.S6 Combined Datasets Immune Infiltration Analysis by CIBERSORT

Fig.S7 IPS Analysis

Algorithm

Table S1 Baseline Table with READ Patients Characteristics

| Characteristics | overall |
| --- | --- |
| Age, mean ± sd | 64.559 ± 11.783 |
| Gender, n (%) |  |
| female. | 75 (46.6%) |
| MALE | 86 (53.4 percent) |
| MStage, n (%) |  |
| M0 | 126 (78.3%) |
| M1 | 23 (14.3%) |
| MX | 12 (7.5%) |
| NStage, n (%) |  |
| N0 | 84 (52.2%) |
| N1 | 44 (27.3%) |
| N2 | 33 (20.5%) |
| TStage, n (%) |  |
| T1&2 | 37 (23%) |
| T3 | 110 (68.3%) |
| T4 | 14 (8.7%) |

READ, rectal Adenocarcinoma.

Table S2 GEO Microarray Chip Information

|  | GSE150082 | GSE35452 |
| --- | --- | --- |
| Platform | GPL13497 | GPL570 |
| Species | Homo sapiens | Homo sapiens |
| Tissue | Rectal | Rectal |
| Samples in Respone group | 16 | 24 |
| Samples in Nonrespone group | 23 | 22 |
| Reference | PMID: 32784964 | \ |

GEO, Gene Expression Omnibus.

Table S3 Results of Cox Analysis

| Characteristics | Total(N) | Univariate analysis | |  | Multivariate analysis | |
| --- | --- | --- | --- | --- | --- | --- |
|  |  | HR (95% CI) | P value |  | HR (95% CI) | P value |
| Age | 161 | 1.088 (1.040-1.137) | < 0.001 |  | 1.051 (0.996-1.110) | 0.072 |
| Gender | 161 |  |  |  |  |  |
| Female. | 75 | Reference |  |  |  |  |
| MALE | 86 | 0.835 (0.374-1.864) | 0.660 |  |  |  |
| MStage | 161 |  |  |  |  |  |
| M0 | 126 | Reference |  |  | Reference |  |
| M1 | 23 | 3.375 (1.412-8.069) | 0.006 |  | 2.499 (0.890-7.022) | 0.082 |
| MX | 12 | 0.537 (0.071-4.083) | 0.548 |  | 1.028 (0.103-10.291) | 0.981 |
| NStage | 161 |  |  |  |  |  |
| N0 | 84 | Reference |  |  | Reference |  |
| N1 | 44 | 2.447 (0.851-7.038) | 0.097 |  | 0.824 (0.247-2.750) | 0.753 |
| N2 | 33 | 3.683 (1.390-9.759) | 0.009 |  | 1.630 (0.427-6.224) | 0.475 |
| TStage | 161 |  |  |  |  |  |
| T1&2 | 37 | Reference |  |  | Reference |  |
| T3 | 110 | 1.161 (0.380-3.544) | 0.793 |  | 0.400 (0.113-1.421) | 0.157 |
| T4 | 14 | 4.302 (1.147-16.130) | 0.030 |  | 0.700 (0.135-3.615) | 0.670 |
| Risk.Score | 161 | 6.855 (3.856-12.187) | < 0.001 |  | 5.398 (2.523-11.550) | < 0.001 |

HR, Hazard ratio, general HR > 1 indicates that the variable is a risk factor, and HR < 1 is a protective factor. Univariate p values < 0.1 were included in the analysis.

Table S4 Result of GO and KEGG Enrichment Analysis for DRMRDEGs

| ONTOLOGY | ID | Description | GeneRatio | | BgRatio | pvalue | p.adjust | qvalue |
| --- | --- | --- | --- | --- | --- | --- | --- | --- |
| BP | GO:0009615 | response to virus | | 14/120 | 392/18800 | 2.10 e-07 | 4.93 e-04 | 4.17 e-04 |
| BP | GO:0002831 | regulation of response to biotic stimulus | | 13/120 | 351/18800 | 3.89 e-07 | 4.93 e-04 | 4.17 e-04 |
| BP | GO:0031532 | actin cytoskeleton reorganization | | 8/120 | 111/18800 | 5.56 e-07 | 4.93 e-04 | 4.17 e-04 |
| BP | GO:0051607 | defense response to virus | | 11/120 | 290/18800 | 2.55 e-06 | 1.40 e-03 | 1.19 e-03 |
| BP | GO:0140546 | defense response to symbiont | | 11/120 | 291/18800 | 2.64 e-06 | 1.40 e-03 | 1.19 e-03 |
| CC | GO:0030055 | cell-substrate junction | | 15/120 | 428/19594 | 5.63 e-08 | 1.77 e-05 | 1.52 e-05 |
| CC | GO:0005925 | focal adhesion | | 14/120 | 419/19594 | 2.87 e-07 | 4.51 e-05 | 3.87 e-05 |
| CC | GO:0005776 | autophagosome | | 6/120 | 112/19594 | 6.56 e-05 | 6.87 e-03 | 5.89 e-03 |
| CC | GO:0030670 | phagocytic vesicle membrane | | 5/120 | 77/19594 | 1.10 e-04 | 8.64 e-03 | 7.42 e-03 |
| CC | GO:0101002 | ficolin-1-rich granule | | 7/120 | 185/19594 | 1.45 e-04 | 9.09 e-03 | 7.80 e-03 |
| KEGG | hsa05215 | Prostate cancer | | 8/79 | 97/8164 | 3.87 e-06 | 4.79 e-04 | 3.10 e-04 |
| KEGG | hsa05230 | Central carbon metabolism in cancer | | 7/79 | 70/8164 | 4.46 e-06 | 4.79 e-04 | 3.10 e-04 |
| KEGG | hsa05160 | Hepatitis C | | 9/79 | 157/8164 | 1.86 e-05 | 1.34 e-03 | 8.63 e-04 |
| KEGG | hsa05226 | Gastric cancer | | 8/79 | 149/8164 | 8.90 e-05 | 4.78 e-03 | 3.09 e-03 |
| KEGG | hsa05169 | Epstein-Barr virus infection | | 9/79 | 202/8164 | 1.34 e-04 | 5.29 e-03 | 3.42 e-03 |

GO, Gene Ontology; BP, Biological Process; CC, Cellular Component; KEGG, Kyoto Encyclopedia of Genes and Genomes; DRMRDEGs, Drug Resistance & Mitophagy-Related Differentially Expressed Genes

Table S5 Results of GSEA for Combined Datasets

| ID | Set Size | Enrichment Score | NES | p value | p.adjust | q value |
| --- | --- | --- | --- | --- | --- | --- |
| WP_IL6_SIGNALING_PATHWAY | 43 | 5.95 e-01 | 2.05 e+00 | 1.92 e-03 | 4.15 e-02 | 3.46 e-02 |
| REACTOME_SHC1_EVENTS_IN_EGFR_SIGNALING | 13 | 7.52 e-01 | 1.95 e+00 | 3.81 e-03 | 6.16 e-02 | 5.14 e-02 |
| REACTOME_FLT3_SIGNALING | 38 | 5.74 e-01 | 1.92 e+00 | 3.88 e-03 | 6.16 e-02 | 5.14 e-02 |
| KEGG_TYPE_I_DIABETES_MELLITUS | 38 | 5.69 e-01 | 1.91 e+00 | 3.88 e-03 | 6.16 e-02 | 5.14 e-02 |
| WP_COVID19_ADVERSE_OUTCOME_PATHWAY | 13 | 7.28 e-01 | 1.89 e+00 | 3.81 e-03 | 6.16 e-02 | 5.14 e-02 |
| BIOCARTA_CTL_PATHWAY | 13 | 7.24 e-01 | 1.88 e+00 | 3.81 e-03 | 6.16 e-02 | 5.14 e-02 |
| REACTOME_AMINE_LIGAND_BINDING_RECEPTORS | 41 | 5.46 e-01 | 1.88 e+00 | 3.77 e-03 | 6.16 e-02 | 5.14 e-02 |
| KEGG_RENIN_ANGIOTENSIN_SYSTEM | 17 | 6.67 e-01 | 1.87 e+00 | 3.88 e-03 | 6.16 e-02 | 5.14 e-02 |
| WP_INTERACTIONS_OF_NATURAL_KILLER_CELLS_IN_PANCREATIC_CANCER | 22 | 6.30 e-01 | 1.86 e+00 | 3.90 e-03 | 6.16 e-02 | 5.14 e-02 |
| WP_IL2_SIGNALING_PATHWAY | 42 | 5.39 e-01 | 1.85 e+00 | 3.86 e-03 | 6.16 e-02 | 5.14 e-02 |
| REACTOME_GPVI_MEDIATED_ACTIVATION_CASCADE | 34 | 5.64 e-01 | 1.85 e+00 | 3.77 e-03 | 6.16 e-02 | 5.14 e-02 |
| REACTOME_SIGNALING_BY_PDGFRA_TRANSMEMBRANE_JUXTAMEMBRANE_AND_KINASE_DOMAIN_MUTANTS | 12 | 7.35 e-01 | 1.85 e+00 | 5.83 e-03 | 7.56 e-02 | 6.30 e-02 |
| REACTOME_SIGNALING_BY_EGFR_IN_CANCER | 24 | 6.12 e-01 | 1.83 e+00 | 3.93 e-03 | 6.17 e-02 | 5.14 e-02 |
| BIOCARTA_EDG1_PATHWAY | 22 | 6.21 e-01 | 1.83 e+00 | 3.90 e-03 | 6.16 e-02 | 5.14 e-02 |
| SIG_CHEMOTAXIS | 44 | 5.26 e-01 | 1.82 e+00 | 3.80 e-03 | 6.16 e-02 | 5.14 e-02 |
| BIOCARTA_PTEN_PATHWAY | 18 | 6.45 e-01 | 1.82 e+00 | 5.88 e-03 | 7.59 e-02 | 6.33 e-02 |
| BIOCARTA_IL2RB_PATHWAY | 37 | 5.47 e-01 | 1.82 e+00 | 3.88 e-03 | 6.16 e-02 | 5.14 e-02 |
| REACTOME_REGULATION_OF_FOXO_TRANSCRIPTIONAL_ACTIVITY_BY_ACETYLATION | 10 | 7.51 e-01 | 1.82 e+00 | 5.70 e-03 | 7.56 e-02 | 6.30 e-02 |
| BIOCARTA_LYM_PATHWAY | 12 | 7.21 e-01 | 1.81 e+00 | 7.77 e-03 | 8.49 e-02 | 7.07 e-02 |
| KEGG_PRIMARY_IMMUNODEFICIENCY | 35 | 5.48 e-01 | 1.81 e+00 | 5.71 e-03 | 7.56 e-02 | 6.30 e-02 |

GSEA, Gene Set Enrichment Analysis.

Table S6 Results of GSVA for Combined Datasets

| ID | logFC | AveExpr | p value | adj.p value |
| --- | --- | --- | --- | --- |
| TOMIDA_LUNG_CANCER_POOR_SURVIVAL | 2.97 e-01 | 6.88 e-03 | 5.05 e-03 | 5.81 e-01 |
| WP_MITOCHONDRIAL_FATTY_ACID_SYNTHESIS_PATHWAY | 2.87 e-01 | 1.74 e-02 | 1.49 e-02 | 6.12 e-01 |
| RANKIN_ANGIOGENIC_TARGETS_OF_VHL_HIF2A_UP | 2.73 e-01 | 1.69 e-02 | 6.36 e-03 | 6.00 e-01 |
| WP_CEREBRAL_ORGANIC_ACIDURIAS_INCLUDING_DISEASES | 2.60 e-01 | 2.82 e-02 | 3.98 e-03 | 5.79 e-01 |
| REACTOME_VITAMINS | 2.55 e-01 | 2.21 e-02 | 9.07 e-03 | 6.01 e-01 |
| WP_FAMILIAL_HYPERLIPIDEMIA_TYPE_1 | 2.50 e-01 | 8.30 e-03 | 5.11 e-04 | 4.78 e-01 |
| REACTOME_UBIQUINOL_BIOSYNTHESIS | 2.47 e-01 | 8.67 e-03 | 1.49 e-02 | 6.12 e-01 |
| REACTOME_NEGATIVE_REGULATION_OF_TCF_DEPENDENT_SIGNALING_BY_DVL_INTERACTING_PROTEINS | 2.38 e-01 | 4.25 e-02 | 1.84 e-02 | 6.12 e-01 |
| BIOCARTA_BARD1_PATHWAY | 2.37 e-01 | 2.58 e-03 | 2.89 e-02 | 6.13 e-01 |
| WP_FAMILIAL_HYPERLIPIDEMIA_TYPE_5 | 2.35 e-01 | 8.77 e-03 | 1.87 e-03 | 5.32 e-01 |
| ROETH_TERT_TARGETS_UP | 2.81 e-01 | 1.70 e-02 | 1.94 e-03 | 5.32 e-01 |
| XU_HGF_TARGETS_INDUCED_BY_AKT1_6HR | 2.82 e-01 | 9.39 e-03 | 7.02 e-04 | 4.78 e-01 |
| KEGG_MEDICUS_REFERENCE_EGF_EGFR_RAS_PI3K_SIGNALING_PATHWAY | 2.94 e-01 | 1.01 e-03 | 6.67 e-04 | 4.78 e-01 |
| MYLLYKANGAS_AMPLIFICATION_HOT_SPOT_29 | 2.94 e-01 | 2.52 e-02 | 2.69 e-03 | 5.37 e-01 |
| KEGG_MEDICUS_REFERENCE_EGF_EGFR_PI3K_SIGNALING_PATHWAY | 3.00 e-01 | 2.45 e-02 | 1.13 e-03 | 4.78 e-01 |
| KEGG_MEDICUS_VARIANT_EGF_OVEREXPRESSION_TO_PI3K_SIGNALING_PATHWAY | 3.01 e-01 | 3.37 e-02 | 1.54 e-03 | 5.20 e-01 |
| MOSERLE_IFNA_RESPONSE | 3.07 e-01 | 6.62 e-03 | 1.41 e-03 | 5.09 e-01 |
| TSAI_DNAJB4_TARGETS_UP | 3.07 e-01 | 2.88 e-02 | 7.30 e-04 | 4.78 e-01 |
| KEGG_MEDICUS_VARIANT_AMPLIFIED_REL_TO_TRANSCRIPTION | 3.20 e-01 | 1.77 e-02 | 1.21 e-03 | 4.78 e-01 |
| KEGG_MEDICUS_VARIANT_IGH_MMSET_FUSION_TO_TRANSCRIPTIONAL_ACTIVATION | 4.88 e-01 | 1.88 e-02 | 1.80 e-03 | 5.32 e-01 |

GSVA, Gene Set Variation Analysis.
